# Supplementary material for: RNA binding of Hfq monomers promotes RelA-mediated hexamerization in a limiting Hfq environment
Source: Nat Commun. 2021 Apr 21;12:2249. doi: 10.1038/s41467-021-22553-x (PMC8060364; doi:10.1038/s41467-021-22553-x)
Supplement: Supplementary file 1 — Supplementary Information [file 41467_2021_22553_MOESM1_ESM.pdf]

Supplementary data

RelA facilitates Hfq assembly to hexamers by interacting with RNA associated with Hfq monomers

Pallabi Basu[1], Maya Elgrably-Weiss[1], Fouad Hassouna[2], Manoj Kumar[2], Reuven Wiener[2] and Shoshy Altuvia[1]*

[1]Department of Microbiology and Molecular Genetics,

[2]Department of Biochemistry and Molecular Biology, IMRIC,

The Hebrew University-Hadassah Medical School, Jerusalem, Israel.

*Corresponding authors: shoshy.altuvia@mail.huji.ac.il

Supplementary Tables

Table S1. List of strains

Lab stock	Relevant genotype	Source
A2	MC4100 Δ(*argF-lac*) U169 *relA1*	Lab collection
A-1063	XTL634 (W3110 *araD<>tetA-sacB-amp*)	(*3*)
A-397	*relA*$^+$	(*4*)
A-398	Δ*relA* Δ*spoT*	(*4*)
A-506	*relA*$^+$ Δ*ryhB*	(*4*)
A-950	*relA*$^+$ Δ*hfq*::*frt*	(*4*)
A-1012	BL21DE3 Δ*hfq*::*cat-sacB*	(*4*)
A-1014	BL21DE3 Δ*hfq*::*cat-sacB* Δ*relA*::*Kan*	(*4*)
A-1036	Δ*relA*::*frt*	This study
A-1046	Δ*relA*::*frt* Δ*ryhB*::*frt*	This study
D-1160	Δ*relA*::*frt* *sodA-lacZ* (λRS552)	This study
D-1161	Δ*relA*::*frt sdhC-lacZ* (λRS552)	This study
D-1172	*relA*C289Y Δ*hfq*::*frt*	This study
D-1182	*relA*C289Y *hfq*$^+$	This study
D-1187	*relA*$^+$ Δ*ryhB sodA-lacZ* (λRS552)	This study
D-1188	Δ*relA*::*frt* Δ*ryhB*::*frt sodA-lacZ* (λRS552)	This study

Table S2. Plasmids

A. General Plasmids

Plasmids	Genetic elements	Origin	Marker	Source or reference
pEF21	PBAD	p15A	Cm^R	(*5*)
pZE12	PL	ColE1	Amp^R	(*6*)
pZE-RyhB	PL-RyhB	ColE1	Amp^R	(*4*)
pET15b	PT7	ColE1	Amp^R	Lab collection
pJO244	PBAD	ColE1	Amp^R	(*5*)
pBOG552	*lacZ* (translation)	pSC101	Kan^R	Lab Collection
pRS552	*lacZ* (translation)	ColE1	Amp^R Kan^R	(*7*)

B. Plasmids constructed in this study

Genetic elements	Construction	Origin	Marker
PBAD-RelA	pEF21 (2530-2345)	p15A	Cm^R
PBAD-RelAΔNTD	PBAD-RelA (2973-2974)	p15A	Cm^R
PBAD-RelAΔCTD	PBAD-RelA (2971-2972)	p15A	Cm^R
PBAD-RelA:C289Y	PBAD-RelA (random mutagenesis)	p15A	Cm^R
PBAD-RelA:C289Y;Y290C	PBAD-RelA:C289Y (3036-3037)	p15A	Cm^R

PBAD-RelA:C289A	PBAD-RelA (3039-3040)	p15A	Cm^R
PBAD-RelA:Q264E	PBAD-RelA (3134-3135)	p15A	Cm^R
PL-RyhBm	PL-RyhB (3266-3267)	ColE1	Amp^R
PT7-RelA	pET15b (2999-3000 and 3031-3032)	ColE1	Amp^R
PT7-RelA:C289Y	PT7-RelA (3069-3070)	ColE1	Amp^R
PT7-RelA:C289Y;Y290C	PT7-RelA (3036-3037)	ColE1	Amp^R
PT7-RelA:C289A	PT7-RelA (3039-3040)	ColE1	Amp^R
PT7-Hfq	pET15b (2687-2351)	ColE1	Amp^R
PT7-Hfq:D9A	PT7-Hfq (3168-3169)	ColE1	Amp^R
PT7-Hfq:K56A	PT7-Hfq (3048-3049)	ColE1	Amp^R
PT7-Hfq:G29A	PT7-Hfq (3170-3171)	ColE1	Amp^R
PT7-Hfq:I30D	PT7-Hfq (3054-3055)	ColE1	Amp^R
PBAD-ChiX	pJO244 (2594-2595)	ColE1	Amp^R
P*nadE-nadE-lacZ*	pBOG552 (2907-2908)	pSC101	Kan^R
P*sdhC-sdhC-lacZ*	pRS552 (2491-2505)	ColE1	$Amp^R Kan^R$
P*sodA-sodA-lacZ*	pRS552 (2927-2928)	ColE1	Amp^R Kan^R

Table S3. List of oligonucleotides

A. Oligonucleotides used for strain construction

Primer	Primer sequence (5'-3')	Use[a]
618	CAA GTG CGA ATG AGA ATG ATT ATT ATT GTC TCG CGA ATG GGA ATT AGC CAT GGT CC	Δ*ryhB*::*cm* (+)
619	GAA CGA AAG ATC AAA AAA AAA GCC AGC AGC GGG CTG GCT TTG AGC GAT TGT AGG CTG GAG CTG C	Δ*ryhB*::*cm* (-)
2187	CGC GTT AAA TAG TTG CGA TTT GCC GAT TTC GGC TGT AGG CTG GAG CTG CTT C	Δ*relA*::*kan* (+)
2188	ATT GTA GAT ACG AGC AAA TTT CGG CCT AAC TCC ATG GGA ATT AGC CAT GGT CC	Δ*relA*::*kan* (-)
2383	GTA CAA ATA AGC ATA TAA GGA AAA GAG AGA ATG GGA ATT AGC CAT GGT CC	Δ*hfq*::*cam* (+)
2384	GCT CCC CGT GTA AAA AAA CAG CCC GAA ACC TGT AGG CTG GAG CTG CTT C	Δ*hfq*::*cm* (-)
3112	TACTGCTTCCGTTACCTCCATCCAACCGAATACAAACGAATTGCCA AACTTCCTAATTTTTGTTGACACTCTATC	*relA*::*tetA-sacB* (+)
3113	CGCAGGCAAATCAACGACATCACCTTTCGGCGTAAAGACGTACAC CCGGTATCAAAGGGAAAACTGTCCATA	*relA*::*tetA-sacB* (-)
3114	GATGCCAGTCTGTTATTGTGGC	*relA*:C289Y (+)
3115	TTCGGCACTCGGCTTATTAAATTG	*relA*:C289Y (-)

[a]Plus (+) and minus (-) strands are indicated.

B. Oligonucleotides used for plasmid construction

Primer	Primer sequence (5'-3')	Use[a]
2530	CCC TGC AGC TGC AAC GCT GGC TCG G	RelA (*Pst*I +)
2345	CCC AAG CTT TAC GAG CAA ATT TCG GC	RelA (*Hind*III -)
2971	TAG GCC GAA ATT TGC TCG	PBAD-RelAΔCTD (WP PCR +)
2972	CAG TTT ACG CAG CCA GG	PBAD-RelAΔCTD (WP PCR -)
2973	ATT GCG TGG CAG GAA GAG	PBAD-RelAΔNTD (WP PCR +)
2974	CAT CGT CCT CTC CTT TAG GG	PBAD-RelAΔNTD (WP PCR -)
3039	GCCGAGCGTTTACAGGATGCCTATGCCGCACTGGGGATAG	RelA:C289A (+)*
3040	ATCCTGTAAACGCTCGGC	RelA:C289A (-)*
3134	CATTTTACGCCAGATGCTG	RelA:Q264E (+)
3135	CAGCATCTGGCGTAAAATG**G**AGAAAAAGAACCTCGC	RelA:Q264E (-)
3036	GCCGAGCGTTTACAGGATTACTGTGCCGCACTGGGGATAGTG CAC	RelA:C289Y;Y290C (+)*
3037	GTAATCCTGTAAACGCTCGGC	RelA:C289Y;Y290C (-)*
2999	GGAGAATCTTTACTTTCAGGGGATGGTTGCGGTAAGAAGTGC	RelA (+)
3000	CTTTGTTAGCAGCCGGATCCTCGAGCTAACTCCCGTGCAACC GACG	RelA (-)
3031	GCACTTCTTACCGCAACCATCCCCTGAAAGTAAAGATTCTCC	pET15b (+)
3032	CGTCGGTTGCACGGGAGTTAGCTCGAGGATCCGGCTGCTAAC AAAG	pET15b (-)
3069	CGTATTGTCGCCGAGCGTTTACAGGATTACTATGCCGCACTG GGGATAG	RelA:C289Y (+)
3070	ATCCTGTAAACGCTCGGCGACAATACG	RelA:C289Y (-)
2687	GGG CCA TGG CTA AGG GGC AAT CTT TAC	Hfq (*Nco*I +)
2351	CGG GAT CCT TAT TCG GTT TCT TCG CTG	Hfq (*BamH*I -)
3048	CAC GGT CAG CCA GAT GGT TTA CGC GCA CGC GAT TTC TAC TGT TG	Hfq:K56A (+)
3049	GTA AAC CAT CTG GCT GAC CGT G	Hfq:K56A (-)
3054	CAG TTT CTA TTT ATT TGG TGA ATG GTG ATA AGC TGC AAG GGC AAA TCG AG	Hfq:I30D (+)
3055	ACC ATT CAC CAA ATA AAT AGA AAC TG	Hfq:I30D (-)
3168	ATGGCTAAGGGGCAATCTTTACAAGCTCCGTTCCTGAACGCA CTGCG	Hfq:D9A (+)
3169	CTTGTAAAGATTGCCCCTTAGCCAT	Hfq:D9A (-)
3170	CCAGTTTCTATTTATTTGGTGAATGCTATTAAGCTGCAAGGGC AAATCG	Hfq:G29A (+)
3171	CATTCACCAAATAAATAGAAACTGG	Hfq:G29A (-)
3266	GTCGTGCTTTCAGGTAGATGGCGAGGGTGAACCTGATCGC	PL-RyhBm (WP PCR +)
3267	ATTGCTCACATTGCTTCCAG	PL-RyhBm (WP PCR -)
2594	CCC GAA TTC ATT AGG TCT TGG CAG TTG	ChiX (*Eco*RI +)
2595	CCC AAG CTT AAA AAA ATG GCC AAT ATC GC	ChiX (*Hind*III -)
2907	GGAA TTC CAT TTT TCA AGC CGC GC	P*nadE-nadE-lacZ* (*Eco*RI +)
2908	CGGGATCC GCC AGC GTC GAA TCC TG	P*nadE-nadE-lacZ* (*Bam*HI -)

2927	GC GAA TTC GTT ACT CAT CTT CTT ATC CTC ATC	P*sodA-sodA-lacZ* (*Eco*RI +)
2928	CG GGA TCC TGA TGG TGT TTG GTG TGG	P*sodA-sodA-lacZ* (*Bam*HI -)
2491	CGG GAA TTC CCC GAC AAA CTA TAT GTA GG	P*sdhC-sdhC-lacZ* (*Eco*RI +)
2505	CGG GAT CCA GAT TAA CAG GTC TTT GTT TTT TCA C	P*sdhC-sdhC-lacZ* (*Bam*HI -)

[a]Plus (+) and minus (-) strands are indicated. Whole plasmid PCR (WP PCR). *These primers were used to construct several plasmids

C. Oligonucleotides used for primer extension and northern analysis

Primer	Primer sequence (5'-3')	Use[a]
470	CTG GAA GCA ATG TGA GC	*ryhB* (N -)
810	GTA GTG CAG GTA ATT CG	*sodB* (PE -)
3203	GCATCGTAAGCATACGGCAGG	*sod*A (PE -)
3273	CACCCGGCTGGCTAAGTAATAC	*ryhB* (PE -)

[a]Plus (+) and minus (-) strands are indicated, N indicates Northern, and PE indicates primer extension

D. Oligonucleotides used for *in-vitro* RNA synthesis

Primer	Primer sequence (5'-3')	Use[a]
567	AAA AAA AAA GCC AGC ACC CGG	RyhB (90 b -)
678	CGA AAT TAA TAC GAC TCA CTA TAG GGA CAG GCG ATC AGG AAG ACC CTC GC	RyhB (90 b +)
3265	TGT GAG CAA TGT CGT GC	RyhB (50 b -)
3272	CGA AAT TAA TAC GAC TCA CTA TAG GGA CAGGCGATCAGGTTCACCCTCGCCATCTACCTG AAAGCACGAC	RyhBm (90 b +)
1764	CGA AAT TAA TAC GAC TCA CTA TAG GGA CAG GGA ACC AAC TGC TTA CGC G	*sodA* (210 b +)
1765	GCG TTG GCG TTG TTT AC	*sodA* (210 b -)
3209	CGAAATTAATACGACTCACTATAGGGACAGG CGACAATACTGGAGATG	*sodA* (56 b +)
3211	GTAAGCATACGGCAGGG	*sodA* (56 b -)
3203	GCATCGTAAGCATACGGCAGG	*sodA* (98 b -)
3198	AGTATTGTCGGGCGGCCGATTG	*sodA*-ΔSD (47 b -)

[a]Plus (+) and minus (-) strands are indicated

E. Oligonucleotides used for real time PCR

Primer	Primer sequence (5'-3')	Use[a]
566	CG CGA TCA GGA AGA CCC	RyhB qRT-PCR (+)
3273	CACCCGGCTGGCTAAGTAATAC	RyhB qRT-PCR (-)
3305	GATTCGTTATCAGTGCAGGAAA	MgrR qRT-PCR (+)
3306	AGTAAACCGGCGGTGAAT	MgrR qRT-PCR (-)
3307	TTTCTGTTGGGCCATTGCAT	MicC qRT-PCR (+)
3308	AAAGCCCGGACGACTGTT	MicC qRT-PCR (-)
3309	TGTATTCGGTCCAGGGAAATG	SraC qRT-PCR (+)
3310	CTGGCGTCGTCATCTATTCTT	SraC qRT-PCR (-)
3313	GGCGCAGAGGAGACAATG	McaS qRT-PCR (+)
3314	TCGACATCCGCCAGACT	McaS qRT-PCR (-)

3301	ATGAGCTATACCCTGCC	*sodA* qRT-PCR (+)
1765	GCG TTG GCG TTG TTT AC	*sodA* qRT-PCR (-)
3302	GGA CCT ACA GAC CAT C	*sdhC* qRT-PCR (+)
3303	CCTTCAGGGGAAGAGAG	*sdhC* qRT-PCR (-)

[a]Plus (+) and minus (-) strands are indicated

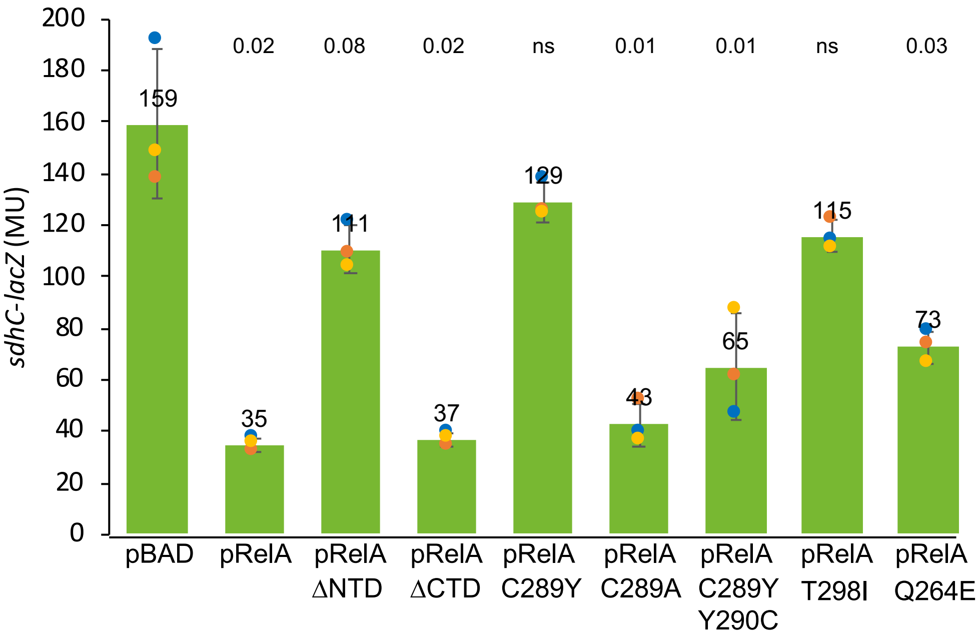

Supplementary Fig. 1 β-galactosidase assay to determine the effect of plasmids encoded RelA alleles on repression of *sdhC-lacZ* target gene fusion by RyhB. Expression of RelA from BAD promoter was induced with (0.2%) arabinose. n=3 biologically independent experiments. Data are presented as mean values +/-SD. Two-tailed unpaired *t*-test were performed; the absolute *P* values are indicated. Source data provided as a source data file.

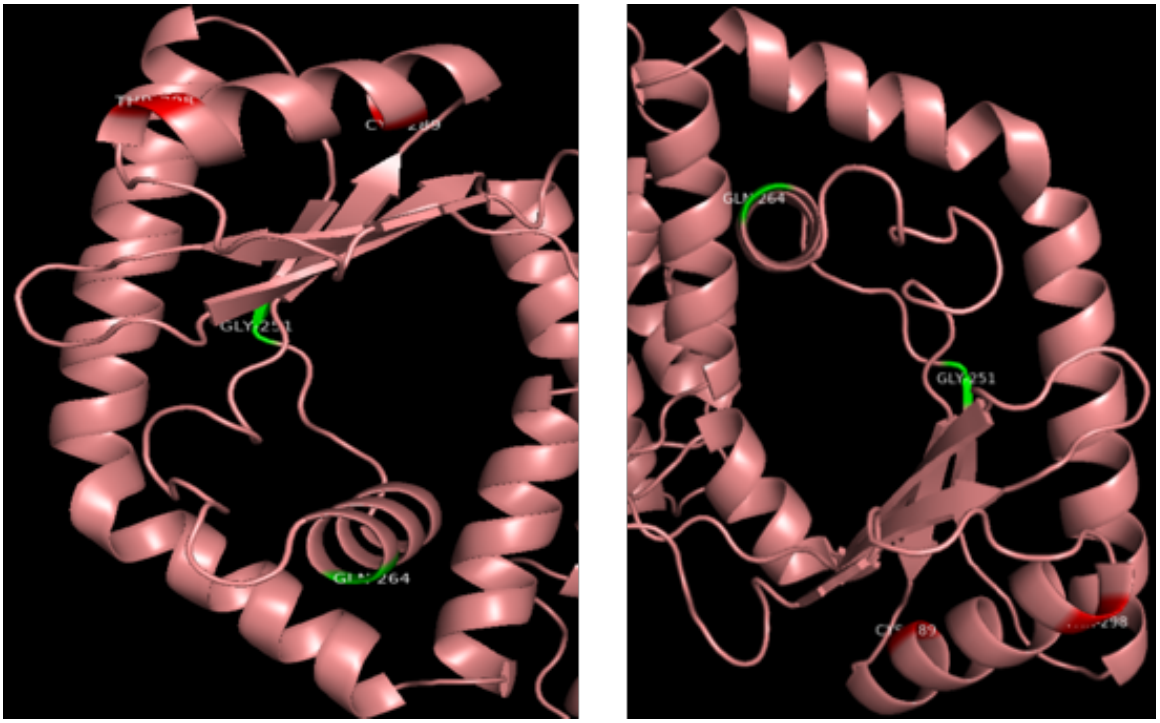

Supplementary Fig. 2 Pymol representation of the amino terminal of RelA. The amino acids marked in red (C289 and T298) are essential for RNA binding activity of RelA, while the amino acids marked in green (G251 and Q264) are essential for the (p)ppGpp synthetic activity of RelA. Presented are two forms of display.

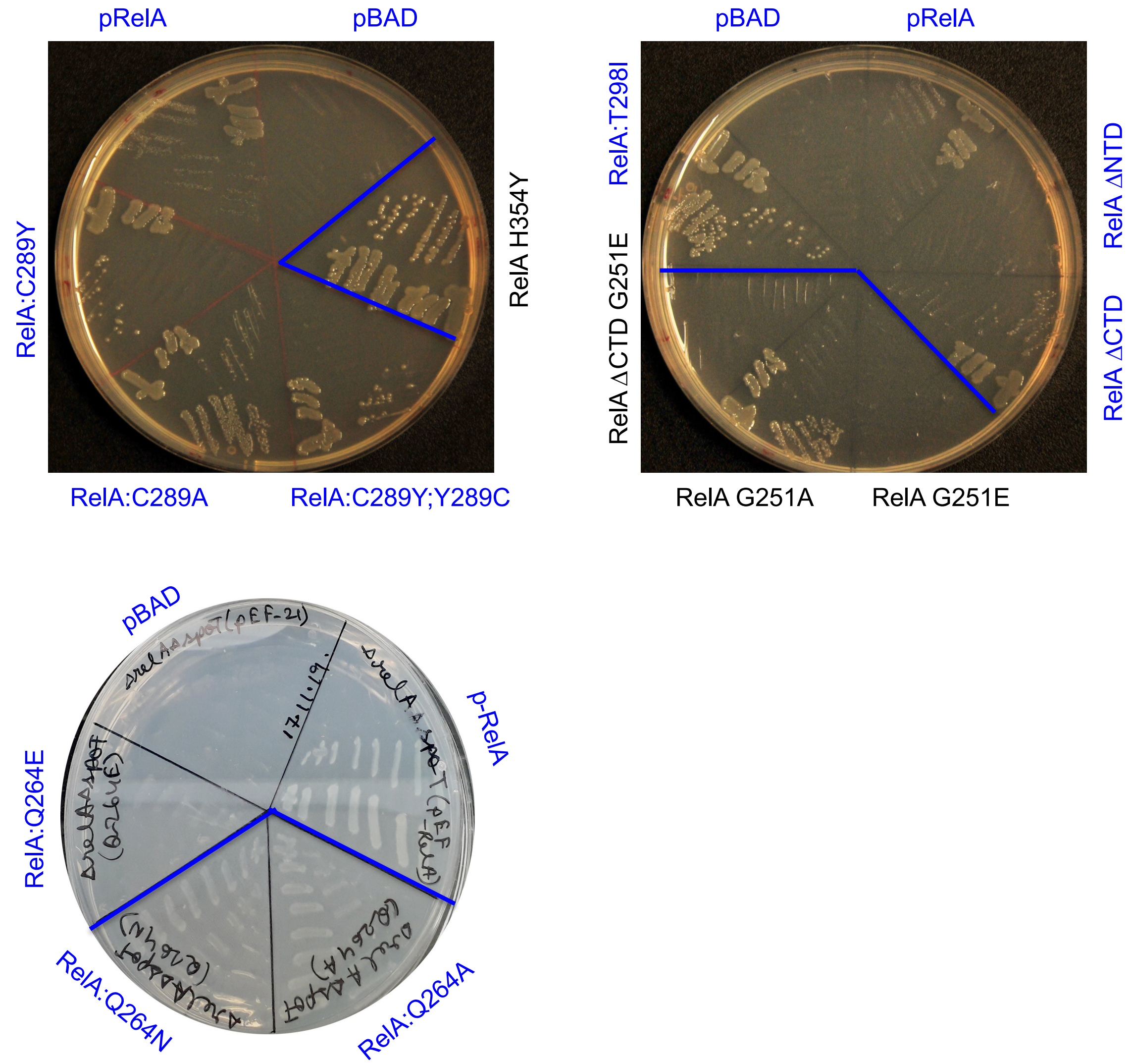

Supplementary Fig. 3 Plasmids expressing RelA enable growth of *E. coli* Δ*relA*Δ*spoT* in M9 minimal medium supplemented with 0.04% glucose, 0.4% glycerol and 0.1% arabinose to induce expression from BAD promoter. The plasmids denoted in blue are relevant for this study. The bottom plate shows the function of *relA* mutants at position Q264. Changing glutamate (Q) to glutamic acid (E) rendered RelA inactive as synthetase, whereas changing Q to alanine (A) or to asparagine (N) had no effect on the activity of RelA as a synthetase.

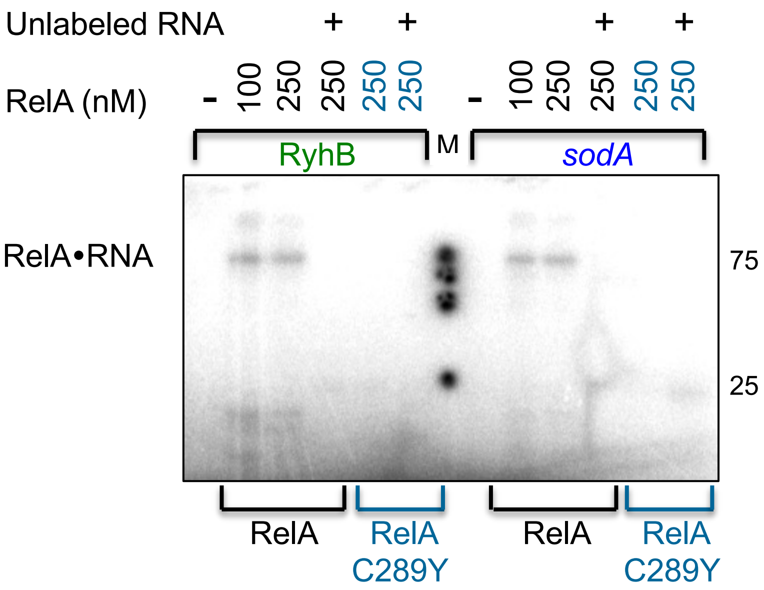

Supplementary Fig. 4 Binding of RyhB and *sodA* by RelA. Wild type RelA (black) or RelA:C289Y (blue) incubated with labeled RyhB (90 nt) or *sodA* (98 nt) RNAs (1 nM) were UV cross-linked. Then, the binding products were treated with 100 μg/ml of RNase A to remove unprotected RNA residues. Competitor unlabeled RNAs (100 nM) was added to the reaction mixtures as indicated. The binding products were analyzed by 15% SDS-PAGE. BLUeye Prestained Protein ladder (MW). The estimated MW of the RelA•RNA complex is ~ 85kDa. Source data provided as a source data file.

RyhB *(E. coli)*
GCGAUCAGGA AGACCCUCGC GGAGAACCUG AAAGCACGAC AUUGCUCACA UUGCUUCCAG
UAUUACUUAG CCAGCCGGGU GCUGGCUUUU

sodA *(E. coli)*
CTGCTTACGC GGCATTAACA ATCGGCCGCC CGACAATACT GGAGATGAAT atgAGCTATA
CCCTGCCATC CCTGCCGTAT GCTTACGATG CCCTGGAACC GCACTTCGAT AAGCAGACCA
TGGAAATCCA CCACACCAAA CACCATCA

sdhC *(E. coli)*
GTCTCCGGAA CACCCTGCAA TCCCGAGCCA CCCAGCGTTG TAACGTGTCG TTTTCGCATC
TGGAAGCAGT GTTTTGCATG ACGCGCAGTT ATAGAAAGGA CGCTGTCTGA CCCGCAAGCA
GACCGGAGGA AGGAAATCCC GACGTCTCCA GGTAACAGAA AGTTAACCTC TGTGCCCGTA
GTCCCCAGGG AATAATAAGA ACAGCATGTG GGCGTTATTC atgATAAGAA ATGTGAAAAA
ACAAAGACCT GTTAATCT

OxyS *(E. coli)*
GAAACGGAGC GGCACCUCUU UUAACCCUUG AAGUCACUGC CCGUUUCGAG AGUUUCUCAA
CUCGAAUAAC UAAAGCCAAC GUGAACUUUU GCGGAUCUCC AGGAUCCGCU

fhlA *(E. coli)*
AGTTAGTCAA TGACCTTTTG CACCGCTTTG CGGTGCTTTC CTGGAAGAAC AAAatgTCAT
ATACACCGAT GAGTGATCTC GGACAACAAG GGTTGTTCGA CATCACTCGG ACACTATTGC
AGCAGCCCGA TCTGGCCTCG CTGTGTGAG

ChiX *(Salmonella)*
ATTAGGTCTT GGCAGTTGCG GCAACTTTGA GCGACAATCT GAAGATCCGA AGCGAAAGCG
TCGGGATAAT AATAACGATG AAATTCCTCT TTGACGGGCC AATAGCGATA TTGGCCA

nadE *(Salmonella)*
GTAAAGATTC ATTTTTTTAA TGTCGAAGGG GGTTAAatgA CTCTGCAGCA AGAGATAATC
CAGGCGCTTG GCGCGAAACC GCATATCAAC CCTGAAGAAG AAATTCGCCG CAGCGTGGAT
TTTCTTAAAG CGTACCTGAA AACCTATCCC TTTTTGAAAT CGCTGGTGTT AGGCATCAGC
GGCGGGCAGG ATTCGACGCT
GGC

ChiX *(E. coli)*
ACACCGUCGC UUAAAGUGAC GGCAUAAUAA UAAAAAAAUG AAAUUCCUCU UUGACGGGCC
AAUAGCGAUA UUGGCCAUUU UUUU

DsrA *(E. coli)*
AACACAUCAG AUUUCCUGGU GUAACGAAUU UUUUAAGUGC UUCUUGCUUA AGCAAGUUUC
AUCCCGACCC CCUCAGGGUC GGGAUUU

Supplementary Fig. 5 Sequences of sRNAs and mRNAs relevant to this study. GGAG site (blue), GGAG variants (purple) RyhB core region (green).

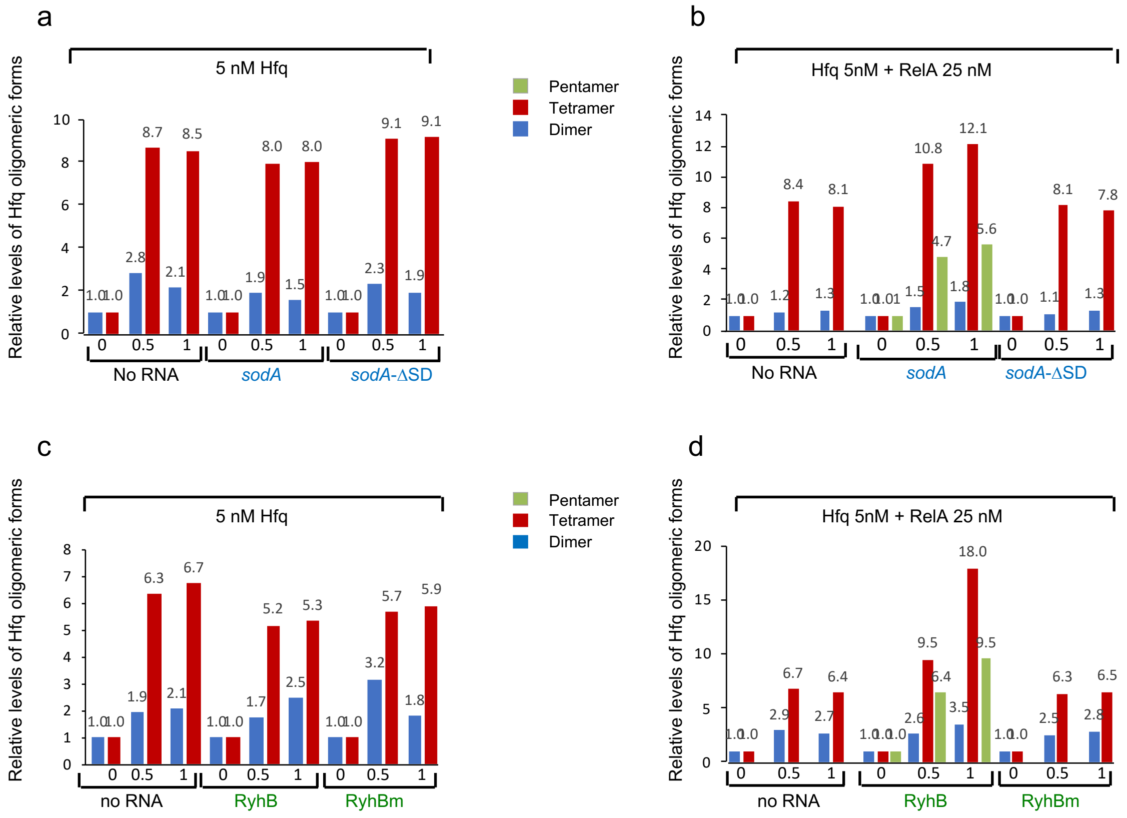

Supplementary Fig. 6 Quantitation of Hfq forms presented in the corresponding panels (a, b, c and d) of Fig. 4. Dimers, tetramers and pentamers were quantitated using ImageLab program. For each set the value measured before cross linking (0 min) served as baseline (1) and the rest were determined accordingly. Source data provided as a source data file.

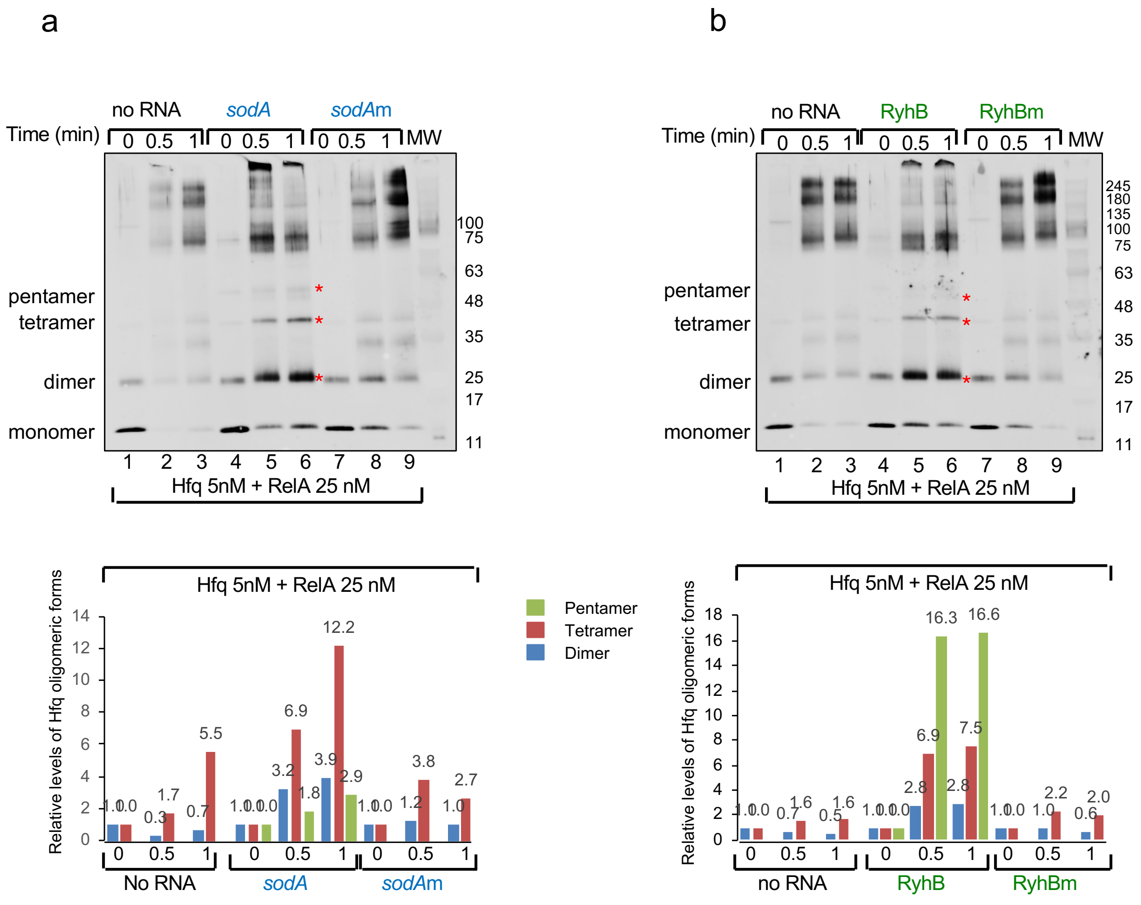

Supplementary Fig. 7 RelA mediated Hfq assembly requires interaction with GGAGA sequence. Reactions of Hfq incubated with RelA, without or with RNA including RyhB, RyhBm (**b**), *sodA* and *sodA*m (**a**) were UV cross-linked followed by protein crosslinking with 0.2% of glutaraldehyde. The proteins separated in 4-20% MOPS gradient gels were detected using a Hfq antibody. BLUeye Prestained Protein ladder (MW). Asterisk indicates the formation of new Hfq multimers detected using wild type RNAs in the presence of RelA. Dimers, tetramers and pentamers were quantitated using ImageLab program. For each set the value measured before cross linking (0 min) served as baseline (1) and the rest were determined accordingly. Source data provided as a source data file.

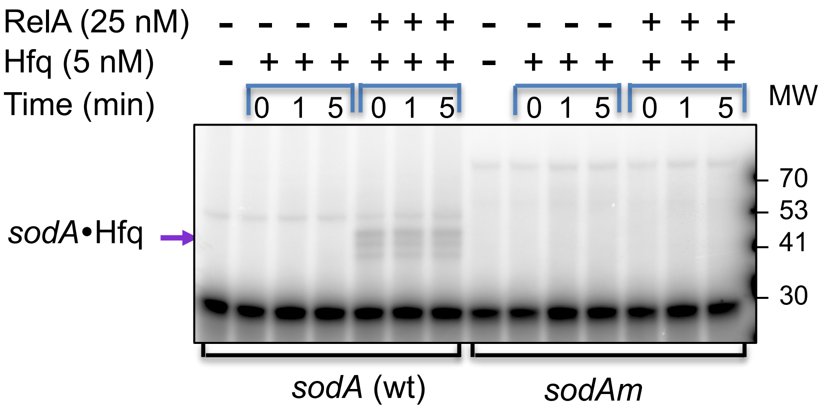

Supplementary Fig. 8 Hfq RNA binding. Reaction mixtures of Hfq incubated for 10 min at 22°C with labeled *sodA* or *sodAm* (1 nM; 98 nt) without or with RelA were UV cross-linked followed by protein crosslinking with 0.2% glutaraldehyde. The cross linking was stopped with 200 mM of fresh glycine and the products analyzed in 4-20% MOPS gradient gel. BLUeye Prestained Protein ladder (MW). RNA bound to one Hfq monomer is indicated by the purple arrow on the left side. Note that the addition of RNA alone to low levels of Hfq (5 nM) does not result in Hfq-RNA stable binding. Also, RelA does not induce binding of RNA lacking GGAGA to Hfq. Source data provided as a source data file.

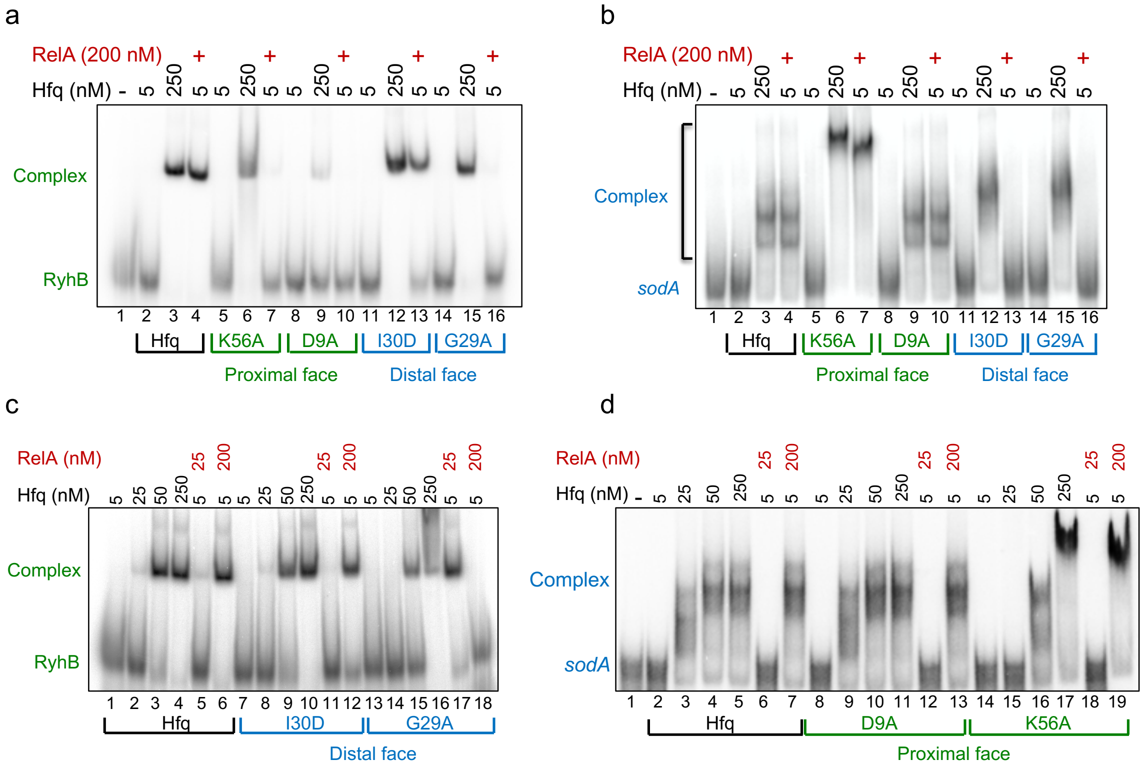

Supplementary Fig. 9 RelA facilitates binding of proximal RNA by Hfq distal mutant and vice versa (EMSA). Wild-type Hfq (black), Hfq proximal mutants K56A and D9A (green) and Hfq distal mutants I30D and G29A (blue) were incubated at 22°C for 10 min. with or without RelA and either (**a and c**) proximal RyhB (50 nt) RNA (green) or (**b and d**) distal *sodA* (201 nt) RNA (blue). The products were separated by 4% native gel electrophoresis. Source data provided as a source data file.

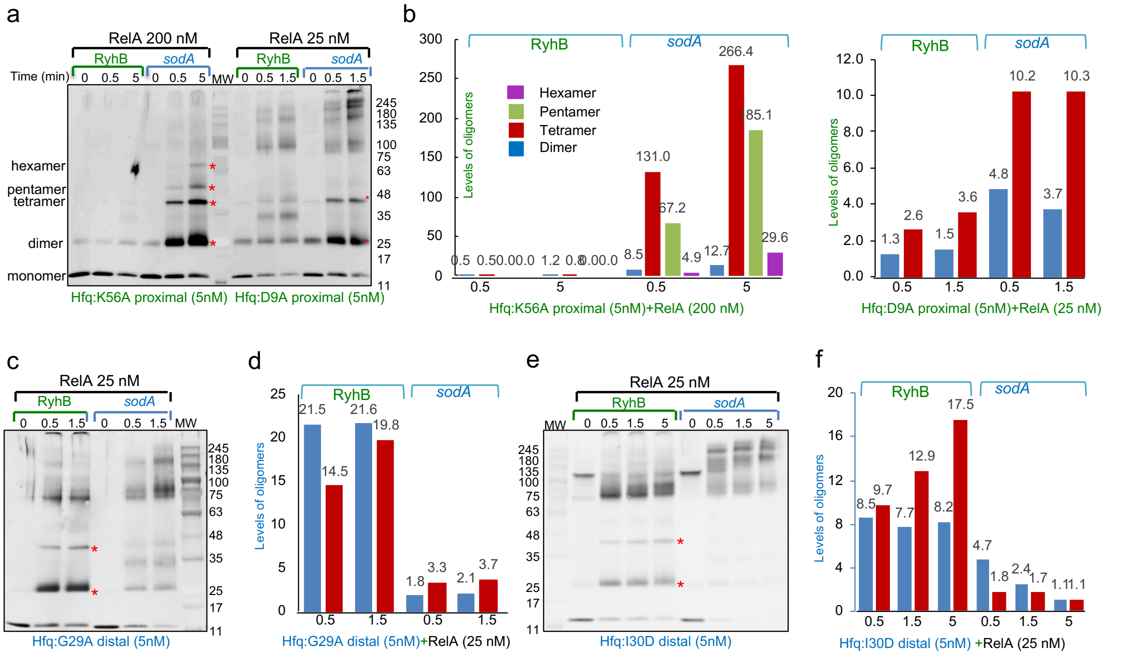

Supplementary Fig. 10 RelA mediated Hfq multimerization requires an initial RNA binding to Hfq (western). Proximal RyhB 90 nt RNA (green) or distal *sodA* 98 nt RNA (blue) were incubated at 22°C for 10 min. with purified RelA protein and with either proximal Hfq mutants Hfq:K56A and Hfq:D9A (green) or with Hfq distal mutants Hfq:G29A and Hfq:I30D (blue). Thereafter, the products were UV cross-linked followed by protein crosslinking using 0.2% glutaraldehyde. Samples were collected at the time points indicated and the reactions stopped with 200 mM of fresh glycine. The proteins separated in 4-20% MOPS gradient gels were detected using a Hfq antibody. BLUeye Prestained Protein ladder (MW). Asterisk denote increased Hfq multimers. Dimers, tetramers and pentamers were quantitated using ImageLab program. For each set the value measured before cross linking (0 min) served as baseline (1) and the rest were determined accordingly. Quantitation of **a,c,e** are presented in **b,d,f**. Source data provided as a source data file.

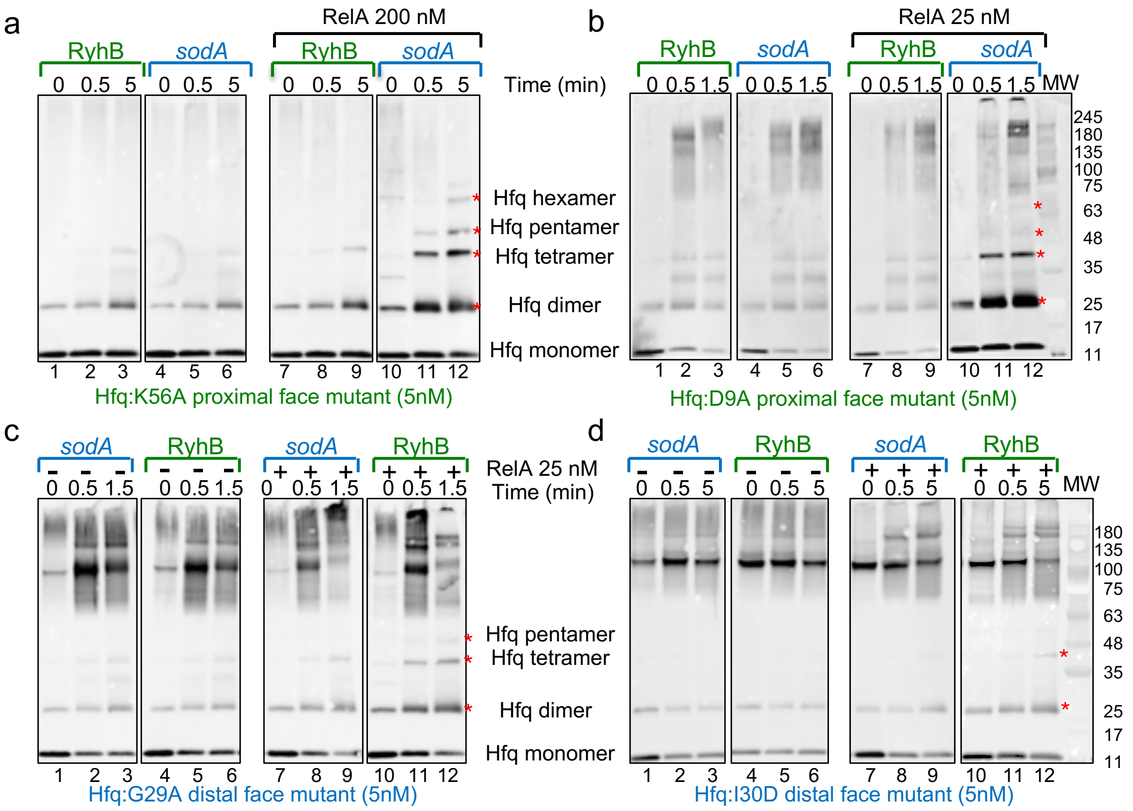

Supplementary Fig. 11 RelA mediated Hfq multimerization requires an initial RNA binding to Hfq (western). Proximal RyhB 90 nt RNA (green) or distal *sodA* 98 nt RNA (blue) were incubated at 22°C for 10 min. with proximal Hfq mutants Hfq:K56A (green; **a**) and Hfq:D9A (green; **b**) or with Hfq distal mutants Hfq:G29A (blue; **c**) and Hfq:I30D (blue; **d**) and with or without purified RelA protein. Thereafter, the products were UV cross-linked followed by protein crosslinking using 0.2% glutaraldehyde. Samples were collected at the time points indicated and the reactions stopped with 200 mM of fresh glycine. The proteins separated in 4-20% MOPS gradient gels were detected using a Hfq antibody. BLUeye Prestained Protein ladder (MW). New/enhanced Hfq multimers are denoted by asterisk. Note that Hfq distal mutants form these multimers in the presence of RelA and RyhB, whereas Hfq proximal mutants form the multimers in the presence of RelA and *sodA* RNA. See quantitation of the multimeric forms in Figure S12. Source data provided as a source data file.

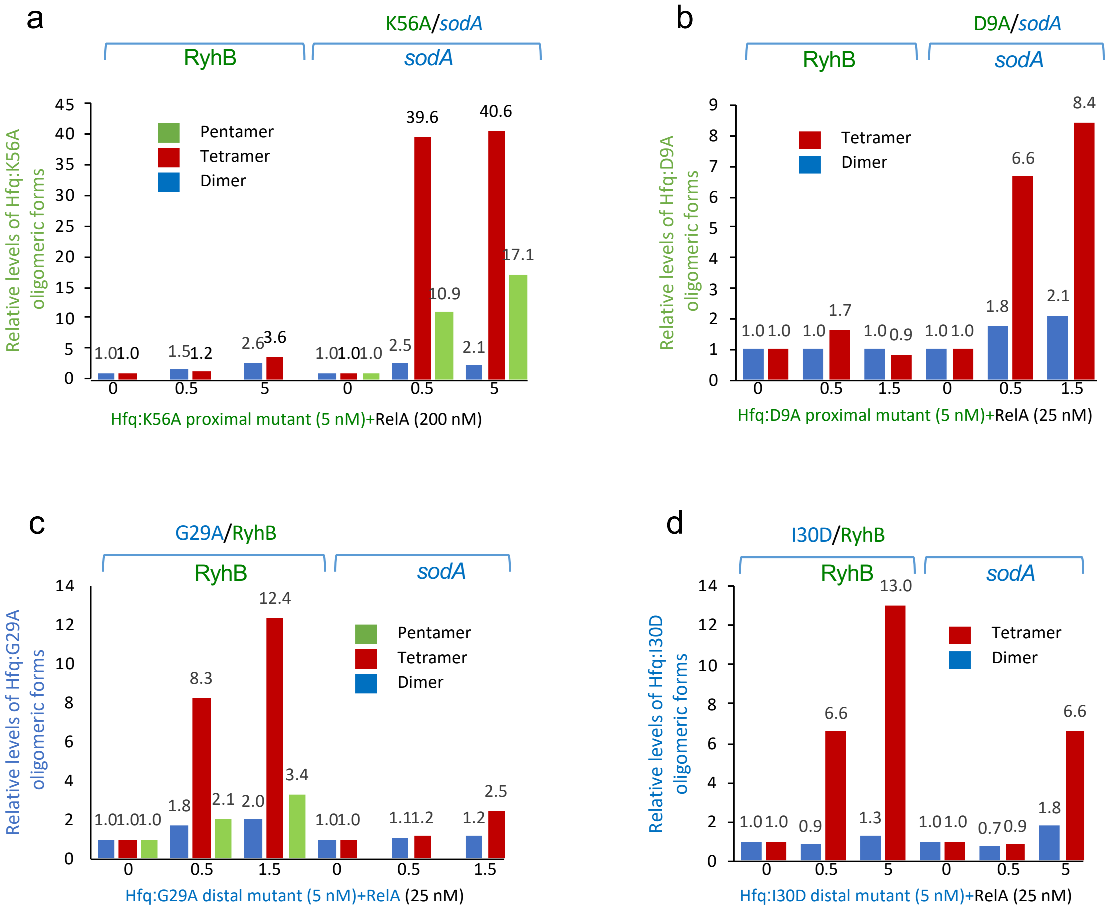

Supplementary Fig. 12 Quantitation of the data presented in the corresponding Figure S11 panels a, b, c and d. Dimers, tetramers and pentamers were quantitated using ImageLab program. For each set the value measured before cross linking (0 min) served as baseline (1) and the rest were determined accordingly. Quantitation was carried out for RelA mediated multimerization of Hfq mutants in the presence of either RyhB or *sodA*. Source data provided as a source data file.

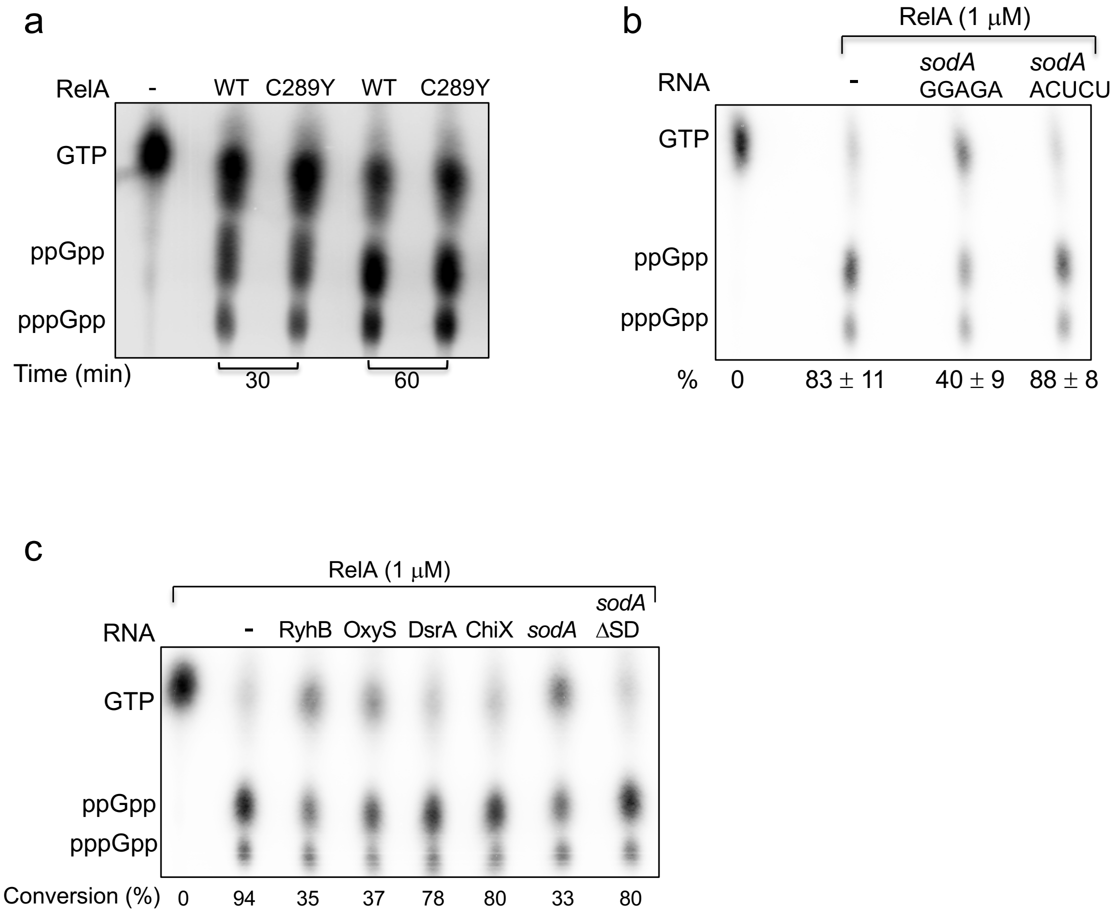

Supplementary Fig. 13 *In-vitro* (p)ppGpp production assays. **a** Purified RelA:C289Y produces (p)ppGpp similar to wild type. Purified RelA wild type (WT) and RelA:C289Y mutant (C289Y) were incubated for the times indicated and ppGpp levels were assayed as describe in Methods. **b** *sodA* carrying an intact GGAGA site inhibits *in-vitro* (p)ppGpp production by RelA. RelA was incubated with 50 nM of RNAs (98 nt) as indicated. Mean of 4 (no RNA or wt *sodA*) and 3 (*sodA* mutants) biological samples. **c** RelA was incubated with 50 nM of either RyhB, OxyS, DsrA, ChiX, *sodA* or *sodA*-ΔSD and assayed by TLC as in Figure 6b. The intensity of the spots was determined by the ImageLab program and percentage of (p)ppGpp production of the total was calculated (% conversion). Source data provided as a source data file.

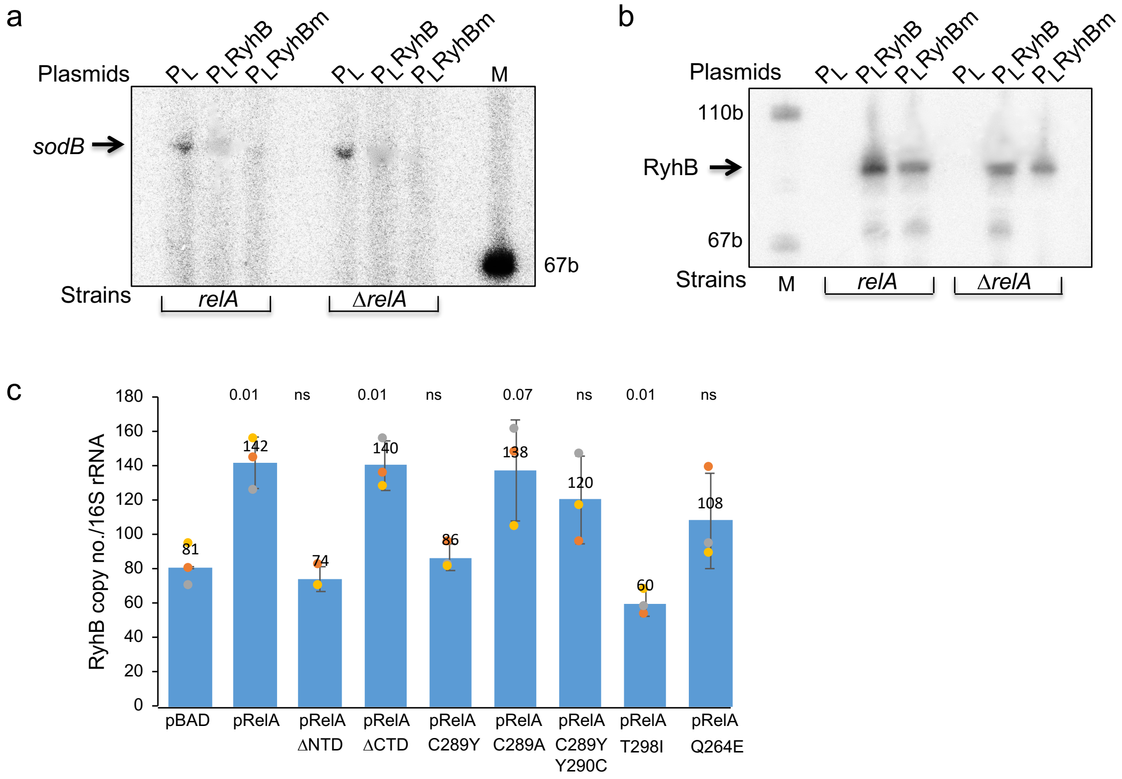

Supplementary Fig. 14 Characterization of RyhB wild type and mutant. **a** Both wild type and RyhBm (lacking GGAGA) repress *sodB*, a RelA-independent target. Primer extension using an *sodB* specific primer carried out with RNA isolated from Δ*ryhB*,*relA*+ and Δ*ryhB*,Δ*relA* strains carrying RyhB expressing plasmids. **b** RelA stabilization of wild type RyhB. RNA as in **a** was analyzed by northern to detect the levels of wild type and RyhB mutant (RyhBm). **c** qRT-PCR of RNA purified from Δ*ryhB* D*relA* cells carrying plasmids expressing RyhB and RelA wild type and mutants as indicated. Duplicates of n=3 biological samples were examined. Data are presented as mean values +/-SD. Two-tailed unpaired *t*-test were performed; the absolute P values are indicated. Source data provided as a source data file.

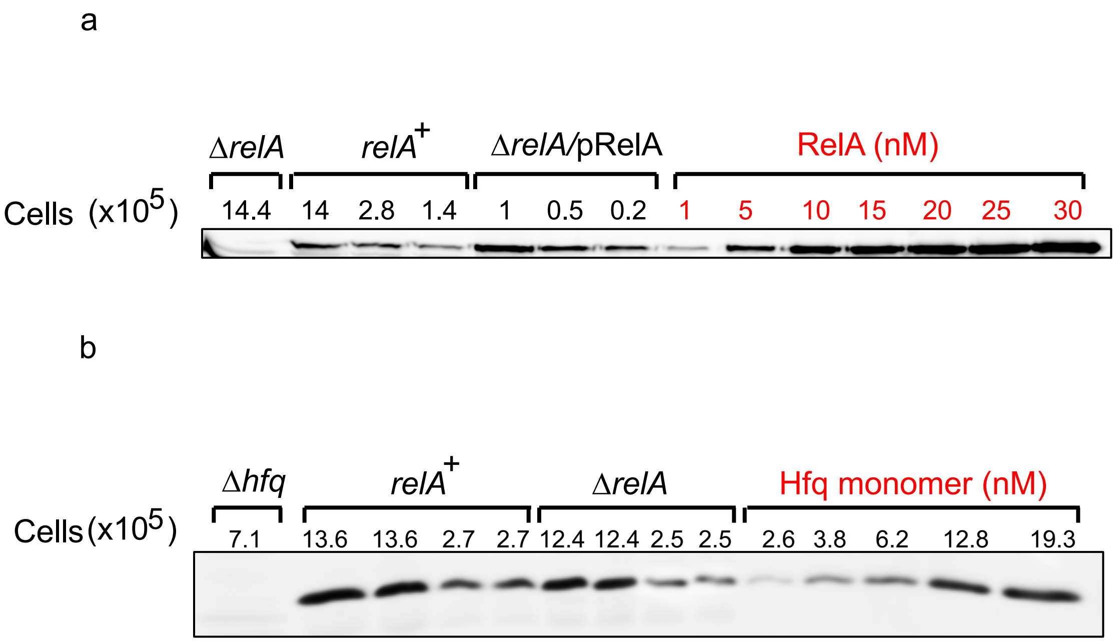

Supplementary Fig. 15 Estimation of (**a**) RelA and (**b**) Hfq concentration. Cultures were grown in 50 ml M9 with glucose (0.04%) and glycerol (0.4%) as described in methods. Cultures carrying pRelA were treated with 0.2% arabinose to induce expression from pRelA. At OD=600 of 0.4, CFU were determined and the cultures collected and resuspended in 1x laemmli sample buffer. The samples were analyzed in 15% SDS-PAGE (RelA) and 4-20% MOPS (Hfq). Chromosomally encoded and plasmid encoded RelA concentrations are 518 ± 150 nM and 2 ± 1 μM, respectively. The concentration of the chromosomally encoded Hfq is ~ 4.5 μM (wild type *relA*) and 2.5 μM (Δ*relA)*, respectively. Given that the lysates showed the presence of Hfq monomers only (see source file), the calculation of the intercellular level of Hfq was determined based on the % of the monomer calculated from the overall concentration of the purified Hfq. Purified Hfq and RelA proteins were used as markers (indicated in red). Source data with MW marker is provided as a source data file.

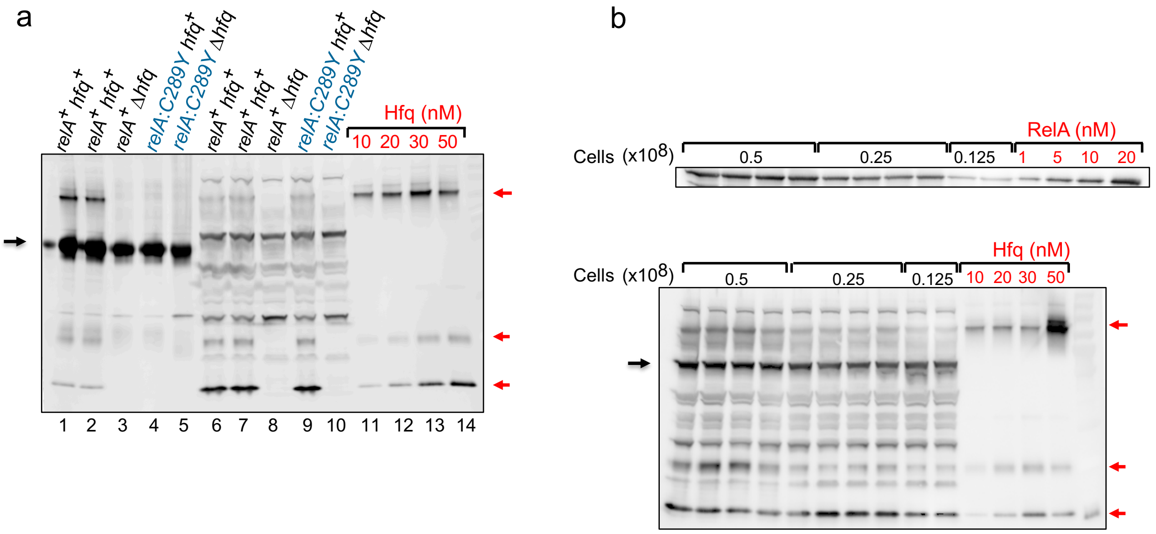

Supplementary Fig. 16 RelA-Hfq *in vivo* complex**. a** Co-immunoprecipitation carried out with a RelA antibody and cell lysates of wild type RelA (black) and RelA:C289Y mutant (blue). Samples were taken after (1-5) and before (6-10) Co-IP. Hfq was detected by western using a Hfq antibody. Purified Hfq was used as control. Red arrows indicate different forms of Hfq. The black arrow indicates the presence of the heavy chain of RelA antibody. Band intensities of Hfq monomers, dimers and dodecamers were measured using ImageLab software and the number of subunits in each form was calculated as compared to the purified Hfq. **b** The number of molecules of RelA and Hfq per cell determined before Co-IP are 4,000 and 15,360 respectively. We estimate that about 50% (± 5) of Hfq can interact with RelA in cell lysates during Co-IP. Mean and SD of two biological samples. Purified Hfq and RelA proteins were used as markers (indicated in red). Source data with MW marker is provided as a source data file.

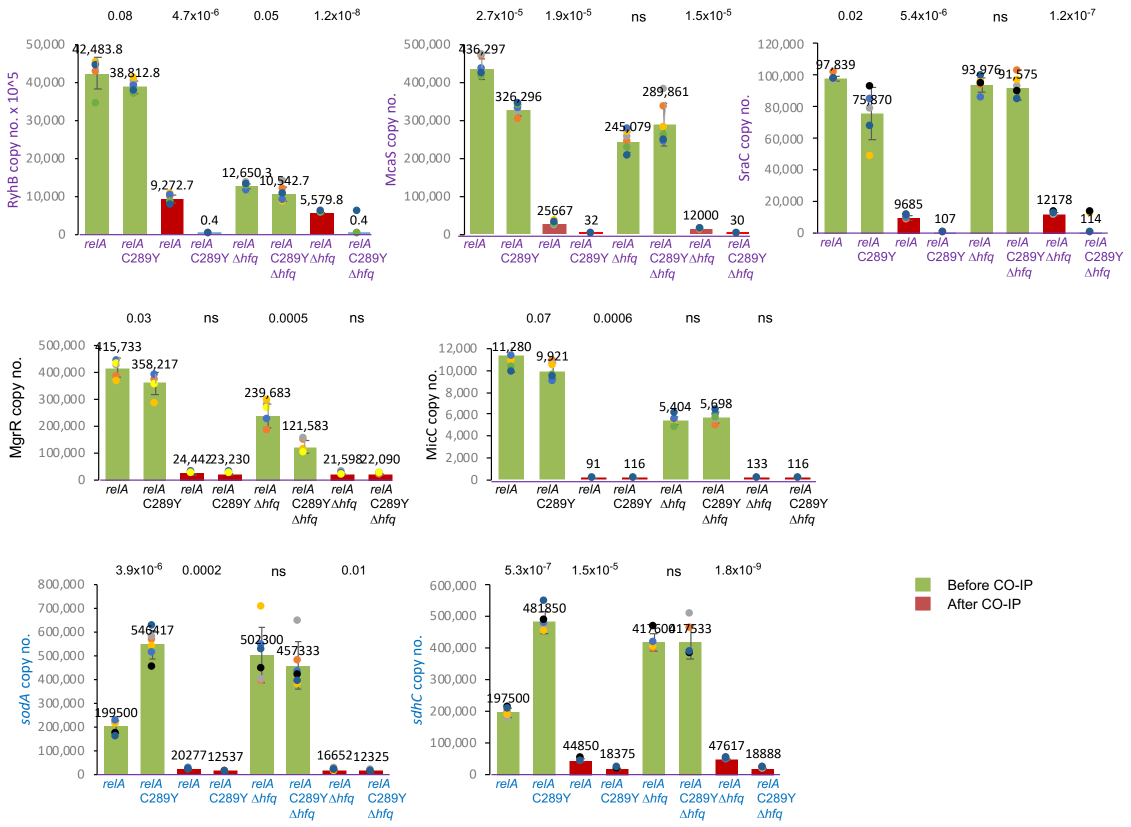

Supplementary Fig. 17 RelA binds sRNAs with GGAG. qRT-PCR of RNA purified from cell lysates as indicated, before (green) and after Co-IP (red). Duplicates of n=3 biological samples were examined. sRNAs with GGAG (purple); sRNAs without GGAG (black); mRNAs with GGAG (blue). Two-tailed unpaired *t*-test were performed; the absolute P values are indicated. Data are presented as mean values +/-SD. Source data provided as a source data file.

sRNA	b-number	GO number	GGAG
3'ETS *leuZ*	b4759	GO-16636	-
4.5S RNA	b0455	EG30027	-
6S RNA	b2911	EG30099	+
AgrA	b4712	GO-10751	-
AgrB	b4713	GO-10752	-
ArcZ	b4450	GO-8871	-
ArrS	b4704	GO-10694	-
ChiX	b4585	GO-9382	-
CpxQ	b4716	GO-16649	-
CsrB	b4408	GO-8785	+
CsrC	b4457	GO-8874	+
CyaR	b4438	GO-8878	-
DicF	b1574	EG31115	-
DsrA	b1954	G7047	-
FnrS	b4699	GO-10677	-
GadF	b4718	GO-16680	-
GadY	b4452	GO-8914	+
GcvB	b4443	GO-8867	-
GlmY	b4441	GO-8910	-
GlmZ	b4456	GO-8873	-
IsrB	b4434	GO-8904	+
IsrC	b4435	GO-8905	-
IstR	b4616	GO-10201	-
McaS	b4426	GO-8899	+
MgrR	b4698	GO-10671	-
MicA	b4442	GO-8866	-
MicC	b4427	GO-8901	-
MicF	b4439	EG30063	-
MicL	b4717	GO-16601	-
OhsC	b4608	GO-10598	-
OmrA	b4444	GO-8868	-
OmrB	b4445	GO-8882	-
OxyS	b4458	EG31116	+
PspH	b4758	GO-16700	-
RalA	b4714	GO-16600	+
RdlA	b4420	GO-9603	-
RdlB	b4422	GO-9606	-
RdlC	b4424	GO-9608	-
RdlD	b4454	GO-9042	-
RirA	b4760	GO-16662	-
RnpB	b3123	EG30069	+
RprA	b4431	GO-8863	-
RseX	b4603	GO-10574	-
RybB	b4417	GO-8880	+

RydB	b4430	GO-8876	+
RydC	b4597	GO-10592	-
RyeA	b4432	GO-8865	+
RyfA	b4440	GO-8879	+
RyfD	b4609	GO-10600	+
RyhB	b4451	GO-8872	+
RyjA	b4459	GO-8875	-
RyjB	b4624	GO-10609	-
SdhX	b4764	GO-17009	-
SdsN	b4719	GO-10699	-
SdsR	b4433	GO-8883	-
SgrS	b4577	GO-9941	-
SibA	b4436	GO-8884	+
SibB	b4437	GO-8885	-
SibC	b4446	GO-8886	+
SibD	b4447	GO-8913	+
SibE	b4611	GO-10602	+
SokB	b4429	GO-9611	-
SokC	b4413	GO-9581	+
SokE	b4700	GO-16677	+
SokX	b4701	GO-10695	-
Spot 42	b3864	EG30098	-
SraA		GO-8861	+
SraB	b4418	GO-8862	-
SraG	b4449	GO-8870	-
SroA	b4762	GO-9381	-
SroC	b4763	GO-9383	-
SroD		GO-9384	-
SroH	b4691	GO-9388	-
SymR	b4625	GO-10610	-
tmRNA	b2621	EG30100	-
XylA-3'UTR	b3565	EG11074	-
aceK internal RNA	b4016	EG10026	+
glnA-3'UTR	b3870	EG10383	+
fadA-3'UTR	b3845	EG10278	-
bhsA-3'UTR	b1112	G6570	-
ybiJ-3'UTR	b0802	EG12422	-
kilR-5'UTR	b1352	EG12155	+
malG-3'UTR (MdoR)	b4032	EG10556	+
rbsB-rbsK IGT	b3751-b3752	EG10815-EG10818	-
allR-3'UTR	b0506	G6276	+

Supplementary Fig. 18 List of sRNAs (*1*, *2*) examined for the presence of GGAG sequence.

a

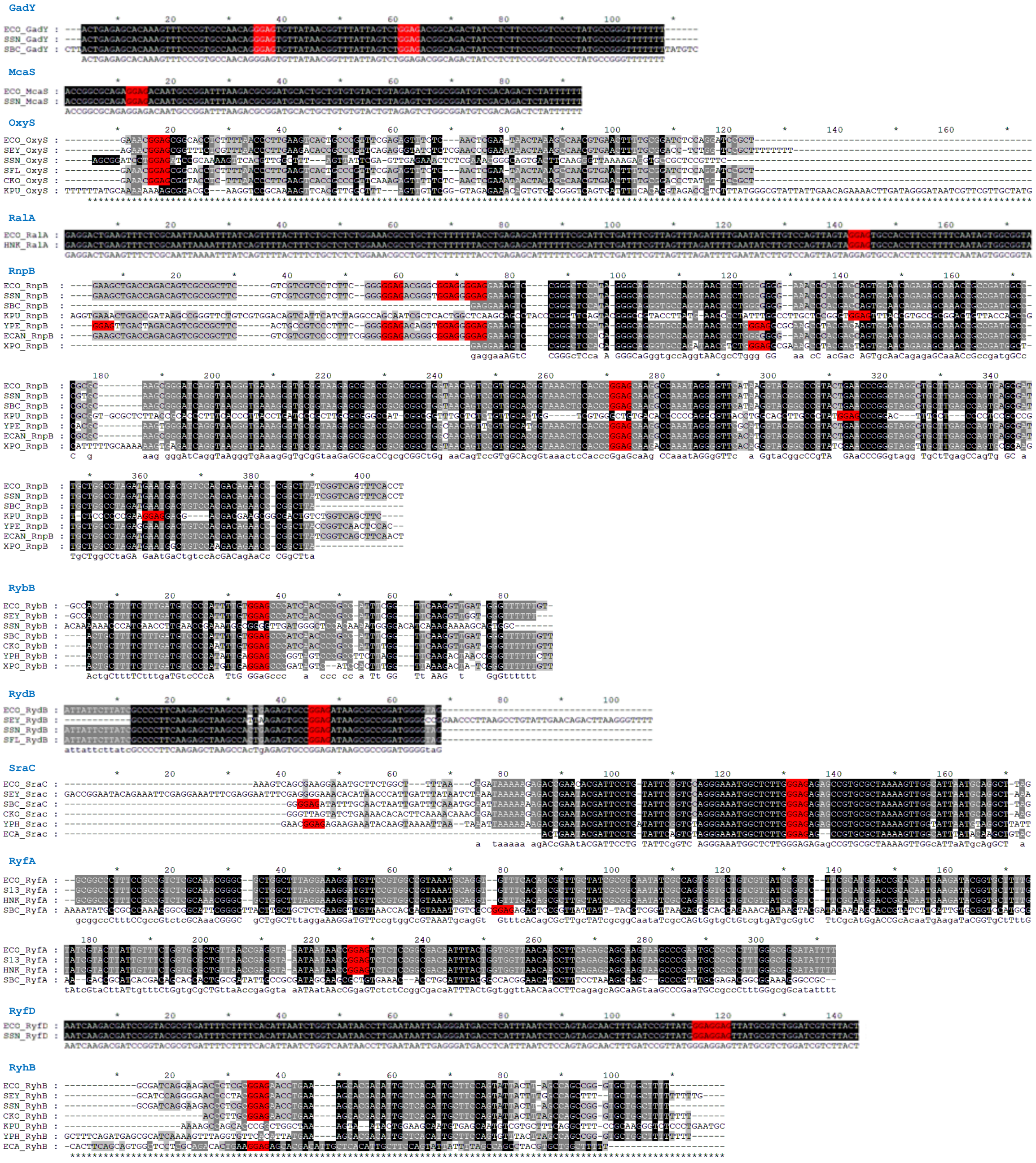

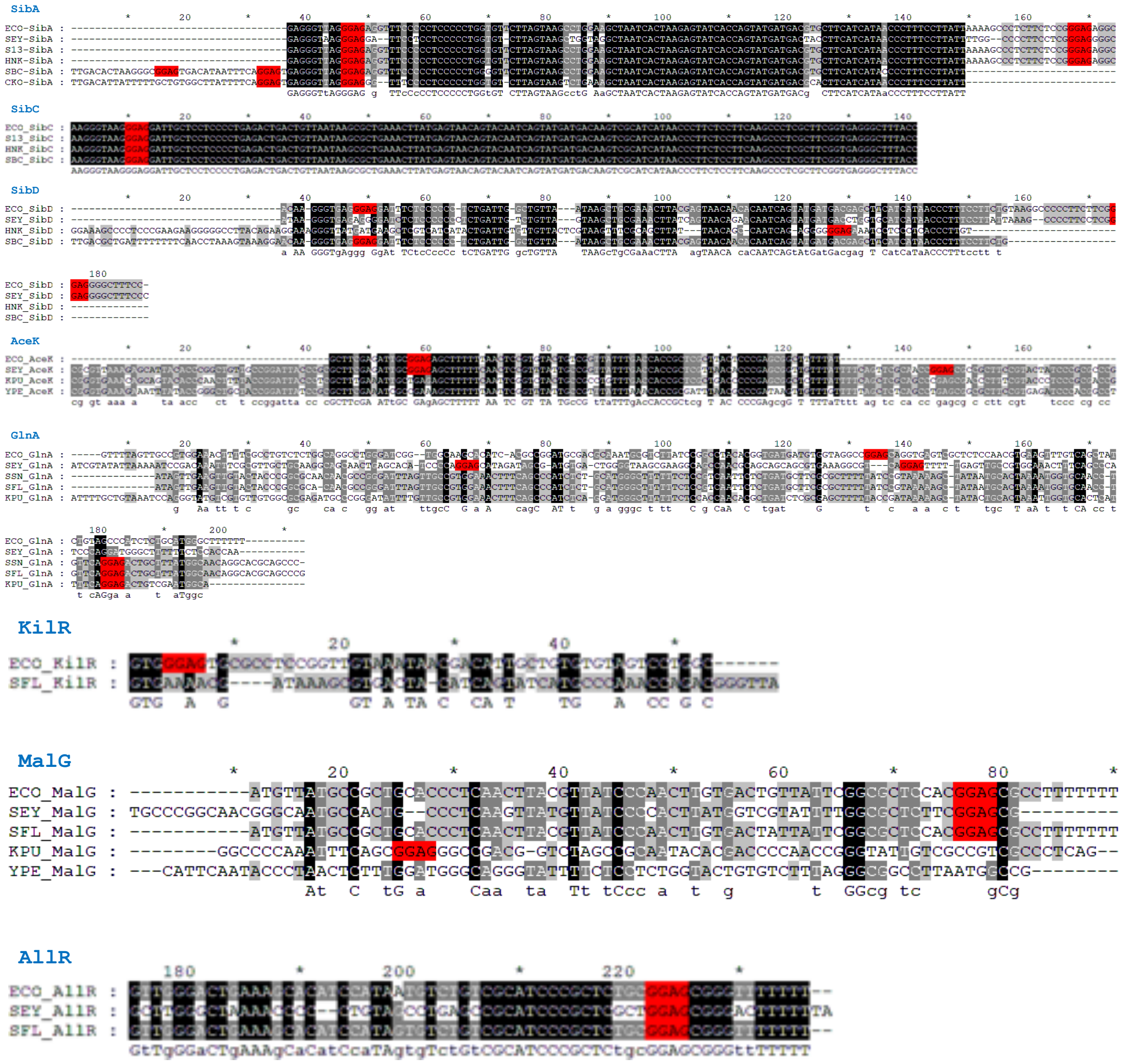
SibA
ECO-SibA
SEY-SibA
S13-SibA
HNK-SibA
SBC-SibA
CKO-SibA
SibC
ECO_SibC
S13_SibC
HNK_SibC
SBC_SibC
SibD
ECO_SibD
SEY_SibD
HNK_SibD
SBC_SibD
AceK
ECO_AceK
SEY_AceK
KPU_AceK
YPE_AceK
GlnA
ECO_GlnA
SEY_GlnA
SSN_GlnA
SFL_GlnA
KPU_GlnA
KilR
ECO_KilR
SFL_KilR
MalG
ECO_MalG
SEY_MalG
SFL_MalG
KPU_MalG
YPE_MalG
AllR
ECO_AllR
SEY_AllR
SFL_AllR

b

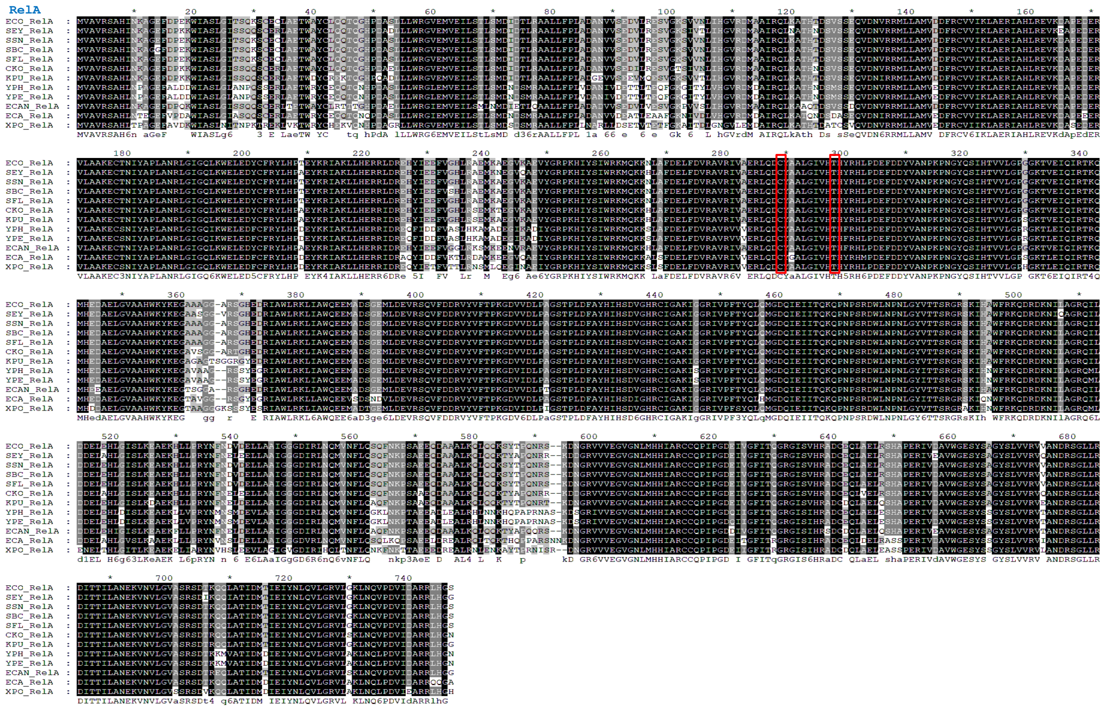

Supplementary Fig. 19. (**a**) Conservation of the GGAG sequence among the sRNAs of diverse enterobacterial species [ECO: *Escherichia coli* K12 MG1655; SEY: *Salmonella enterica subsp*. *Enterica serovar Typhimurium* SL1344; S13: *Salmonella sp*. S13; HNK: *Salmonella sp*. *HNK130*; SSN: *Shigella sonnei*; SBC: *Shigella boydii*; SFL: *Shigella flexneri*; CKO: *Citrobacter koseri*; KPU: *Klebsiella pneumoniae subsp*. *Pneumoniae* NTUH-K2044 (serotype K1); ECA: *Pectobacterium atrosepticum* SCRI1043; ECAN: *Enterobacter cancerogenus*; YPH: *Yersinia pestis Harbin* 35; YPE: *Yersinia pestis* CO92 (biovar Orientalis) and XPO: *Xenorhabdus poinarii*]. Nucleotides in the black regions indicate complete conservation (also indicated by uppercase letters at the bottom of the sequences) while nucleotides in the grey regions indicate partial conservation (also indicated by lowercase letters at the bottom of the sequences). The GGAG sequence is highlighted in red. (**b**) Conservation of RelA amino acid sequence among diverse enterobacterial species (see next slide) [ECO: *Escherichia coli* K12 MG1655; SEY: *Salmonella enterica subsp*. *Enterica serovar Typhimurium* SL1344; S13: *Salmonella sp*. S13; HNK: *Salmonella sp*. *HNK130*; SSN: *Shigella sonnei*; SBC: *Shigella boydii*; SFL: *Shigella flexneri*; CKO: *Citrobacter koseri*; KPU: *Klebsiella pneumoniae subsp*. *Pneumoniae* NTUH-K2044 (serotype K1); ECA: *Pectobacterium atrosepticum* SCRI1043; ECAN: *Enterobacter cancerogenus*; YPH: *Yersinia pestis Harbin* 35; YPE: *Yersinia pestis* CO92 (biovar Orientalis) and XPO: *Xenorhabdus poinarii*]. Nucleotides in the black regions indicate complete conservation (also indicated by uppercase letters at the bottom of the sequences) while nucleotides in the grey regions indicate partial conservation

(also indicated by lowercase letters at the bottom of the sequences). The amino acids at position 289 (C289) and 298 (T298) are marked by red boxes.

Bibliography

1. J. Hör, G. Matera, J. Vogel, S. Gottesman, G. Storz, Trans-Acting Small RNAs and Their Effects on Gene Expression in. *EcoSal Plus* **9**, (2020).
2. S. Melamed *et al.*, Global Mapping of Small RNA-Target Interactions in Bacteria. *Mol Cell* **63**, 884-897 (2016).
3. X. T. Li, L. C. Thomason, J. A. Sawitzke, N. Costantino, D. L. Court, Positive and negative selection using the tetA-sacB cassette: recombineering and P1 transduction in Escherichia coli. *Nucleic Acids Res* **41**, e204 (2013).
4. L. Argaman, M. Elgrably-Weiss, T. Hershko, J. Vogel, S. Altuvia, RelA protein stimulates the activity of RyhB small RNA by acting on RNA-binding protein Hfq. *Proc Natl Acad Sci U S A* **109**, 4621-4626 (2012).
5. L. M. Guzman, D. Belin, M. J. Carson, J. Beckwith, Tight regulation, modulation, and high-level expression by vectors containing the arabinose PBAD promoter. *J Bacteriol* **177**, 4121-4130 (1995).
6. R. Lutz, H. Bujard, Independent and tight regulation of transcriptional units in Escherichia coli via the LacR/O, the TetR/O and AraC/I1-I2 regulatory elements. *Nucleic Acids Res* **25**, 1203-1210 (1997).
7. R. W. Simons, F. Houman, N. Kleckner, Improved single and multicopy lac-based cloning vectors for protein and operon fusions. *Gene* **53**, 85-96 (1987).
