# Supplementary material for: RNA binding of Hfq monomers promotes RelA-mediated hexamerization in a limiting Hfq environment
Source: Nat Commun. 2021 Apr 21;12:2249. doi: 10.1038/s41467-021-22553-x (PMC8060364; doi:10.1038/s41467-021-22553-x)
Supplement: Supplementary file 3 — Reporting Summary [file 41467_2021_22553_MOESM3_ESM.pdf]

nature research

Corresponding author(s): Shoshy Altuvia

Last updated by author(s): Mar 10, 2021

Reporting Summary

Nature Research wishes to improve the reproducibility of the work that we publish. This form provides structure for consistency and transparency in reporting. For further information on Nature Research policies, see our Editorial Policies and the Editorial Policy Checklist.

Statistics

For all statistical analyses, confirm that the following items are present in the figure legend, table legend, main text, or Methods section.

n/a	Confirmed	
☐	☒	The exact sample size (*n*) for each experimental group/condition, given as a discrete number and unit of measurement
☐	☒	A statement on whether measurements were taken from distinct samples or whether the same sample was measured repeatedly
☐	☒	The statistical test(s) used AND whether they are one- or two-sided<br>*Only common tests should be described solely by name; describe more complex techniques in the Methods section.*
☒	☐	A description of all covariates tested
☒	☐	A description of any assumptions or corrections, such as tests of normality and adjustment for multiple comparisons
☐	☒	A full description of the statistical parameters including central tendency (e.g. means) or other basic estimates (e.g. regression coefficient) AND variation (e.g. standard deviation) or associated estimates of uncertainty (e.g. confidence intervals)
☐	☒	For null hypothesis testing, the test statistic (e.g. *F*, *t*, *r*) with confidence intervals, effect sizes, degrees of freedom and *P* value noted<br>*Give P values as exact values whenever suitable.*
☒	☐	For Bayesian analysis, information on the choice of priors and Markov chain Monte Carlo settings
☒	☐	For hierarchical and complex designs, identification of the appropriate level for tests and full reporting of outcomes
☒	☐	Estimates of effect sizes (e.g. Cohen's *d*, Pearson's *r*), indicating how they were calculated

Our web collection on statistics for biologists contains articles on many of the points above.

Software and code

Policy information about availability of computer code

Data collection	IDT PrimerQuest software (https://eu.idtdna.com/PrimerQuest/Home/Index?Display=SequenceEntry)
Data analysis	IDT OligoAnalyzer software (http://eu.idtdna.com/analyzer/Applications/OligoAnalyzer/), Rotor-gene analysis software 6.0, Image Quant LAS 4000 mini software, ImageLab software, Clustal omega (https://www.ebi.ac.uk/Tools/msa/clustalo/) and Genedoc software

For manuscripts utilizing custom algorithms or software that are central to the research but not yet described in published literature, software must be made available to editors and reviewers. We strongly encourage code deposition in a community repository (e.g. GitHub). See the Nature Research guidelines for submitting code & software for further information.

Data

Policy information about availability of data

All manuscripts must include a data availability statement. This statement should provide the following information, where applicable:

- Accession codes, unique identifiers, or web links for publicly available datasets
- A list of figures that have associated raw data
- A description of any restrictions on data availability

Authors can confirm that all relevant data are included in the paper and/or its supplementary information files. Source data are provided with this paper. Following is the list of figures that have associated raw data Fig.1,2,3, 4,5,6 and Supplementary Figure 4,7,8,9,10,11,13,14,15,16 as well as an Excel file with the calculations for all the Bar diagrams. The list of sRNAs provided in supplementary figure 18 was obtained from Biocyc.org with their b-number and GO-number as provided.

Field-specific reporting

Please select the one below that is the best fit for your research. If you are not sure, read the appropriate sections before making your selection.

☒ Life sciences ☐ Behavioural & social sciences ☐ Ecological, evolutionary & environmental sciences

For a reference copy of the document with all sections, see nature.com/documents/nr-reporting-summary-flat.pdf

Life sciences study design

All studies must disclose on these points even when the disclosure is negative.

Sample size	Figures with statistical calculations for lacZ assay involved the use of 3-5 bacterial colonies to check expression and regulation. For real time PCR, RNA was isolated from 3 bacterial colonies and expression was determined with duplicates of each colony. Standard deviation obtained based on the results from 3-5 colonies was low indicating more accurate results due to less variation.
Data exclusions	no data was excluded
Replication	For statistical calculation involving mean and standard deviation, 3-5 independent experiments were done and 2 independent experiments were done for figures involving gels and micrographs.
Randomization	Randomization was not done as samples were not allocated to separate experimental groups or clinical trials.
Blinding	Blinding was not done as samples were not allocated to separate experimental groups or clinical trials.

Reporting for specific materials, systems and methods

We require information from authors about some types of materials, experimental systems and methods used in many studies. Here, indicate whether each material, system or method listed is relevant to your study. If you are not sure if a list item applies to your research, read the appropriate section before selecting a response.

Materials & experimental systems

n/a	Involved in the study	
☐	☒	Antibodies
☒	☐	Eukaryotic cell lines
☒	☐	Palaeontology and archaeology
☒	☐	Animals and other organisms
☒	☐	Human research participants
☒	☐	Clinical data
☒	☐	Dual use research of concern

Methods

n/a	Involved in the study	
☒	☐	ChIP-seq
☒	☐	Flow cytometry
☒	☐	MRI-based neuroimaging

Antibodies

Antibodies used	Rabbit anti-Hfq [raised against a synthetic peptide (SSAQNTSAQQDSEETE) of Hfq CTD (HY-LABS)] and rabbit anti-RelA antibody raised against the purified RelA protein ADAR BIOTECH. Proteins purified by us was used to generate the primary antibody. It is not available commercially. Primary antibody dilutions used is 1:500. Goat anti-rabbit HRP conjugated secondary antibody, Abcam, ab6721. Secondary antibody dilution used is 1:10000.
Validation	The antibodies were tested with purified Hfq and RelA proteins as observed in the western blot figures in the manuscript.
