# Supplementary material for: RNA binding of Hfq monomers promotes RelA-mediated hexamerization in a limiting Hfq environment
Source: Nat Commun. 2021 Apr 21;12:2249. doi: 10.1038/s41467-021-22553-x (PMC8060364; doi:10.1038/s41467-021-22553-x)
Supplement: Supplementary file 4 — Source Data [file 41467_2021_22553_MOESM4_ESM.pdf]

Fig. 1a

Miller Units

						Mean	S.D.	p-value (t-test)
sodA(pBAD)	2775.974	2878.229	2279.152	2491.961	2559.122	2596.888	236.9687033	
sodA(pRelA)	225	285.627	343.590	336.257	414.079	320.911	70.48757613	5.72567E-05
sodA(pRelAΔNTD)	2016.129	2126.736	1442	1459.948	1482.188	1705.400	336.721728	0.000201892
sodA(pRelAΔCTD)	440.158	229	284.853	280.475	324.454	311.788	79.37126492	2.81106E-05
sodA(pRelAC289Y)	2368.805	2054.455	1864.111	1594.005	1652.685	1906.812	316.0253606	0.003856328
sodA(pRelAC289A)	526.316	551.282	429.142	755.926	444.444	541.422	130.7180995	5.5307E-05
sodA(pRelAC289YY290C)	875.113	819.856	985.600	403.646	450.450	706.933	262.8962804	0.000250111
sodA(pRelAT298I)	1851.585	1644.543	1367.596	1411.846	1407.363	1536.587	207.1806663	7.31206E-05
sodA(pRelAQ264E)	518.868	497.010	620.063	579.545	524.617	548.021	50.45693685	8.7408E-05

Fig. 6c

without SHMT

				Mean	SD	t-test (p value)
relA^{+}pP_L	195.499	195.002	200.094162	196.865	2.80736	
relA^{+}pLRyhB	62.5864	65.8078	58.13172043	62.175	3.854519	2.77027E-06
relA^{+}pLRyhBm	181.509	170.977	181.4285714	177.971	6.057421	0.018733579
relA^{-}pP_L	205.058	201.172	214.9470899	207.059	7.102309	
relA^{-}pLRyhB	188.073	194.444	179.7583082	187.425	7.364482	0.02933339
relA^{-}pLRyhBm	183.435	198.351	181.7738791	187.853	9.129361	0.048474633

with SHMT

relA^{+}pP_L	171.032	178.947	178.571	176.184	4.465167	
relA^{+}pLRyhB	174.580	180.717	167.169	174.155	6.784158	0.690870714
relA^{+}pLRyhBm	165.425	170.577	163.451	166.484	3.679007	0.045905014
relA^{-}pP_L	194.190	213.441	175.312	194.314	19.06464	
relA^{-}pLRyhB	175.955	190.686	171.750	179.463	9.944028	0.317179242
relA^{-}pLRyhBm	204.352	233.440	186.476	208.090	23.70384	0.478556388

Fig. 6d

				Mean	SD	t-test (p value)
**relA (pBAD) -SHMT**	2960.663	2632.653	2919.192	2838.000	178.6125	
**relA (pChiX) - SHMT**	753.871	699.923	680.354	711.000	38.07474	0.002080809
**relA (pBAD)+SHMT**	938.889	890.023	1084.025	971.000	100.9036	
**relA (pChiX)+SHMT**	223.799	237.654	298.463	253.000	39.71661	0.002861215

Fig. 6e

				Mean	SD	t-test(p-value)
**pPlacO - SHMT**	20.28	22.109	23.820	22.069	1.77106	
**pOxyS - SHMT**	13.11	14.541	10.699	12.774	1.958001	0.040
**pPlacO + SHMT**	21.89	19.286	20.132	20.436	1.328001	
**pOxyS + SHMT**	16.65	18.775	21.821	19.082	2.599577	0.576

Fig. S1

				Mean	S.D.	t test (pvalue)
**sdhC(pBAD)**	137.931	191.761	147.754	159.1487	28.66699	
**sdhC(pRelA)**	32.216	37.342	35.28	34.946	2.57927	0.016672
**sdhC(pRelAΔNTD)**	108.242	120.532	102.927	110.567	9.029852	0.087851
**sdhC(pRelAΔCTD)**	34.113	39.38	36.863	36.78533	2.634359	0.017144
**sdhC(pRelAC289Y)**	124.688	137.603	123.698	128.663	7.758075	0.201105
**sdhC(pRelAC289A)**	52.031	39.201	36.567	42.59967	8.273272	0.014009
**sdhC(pRelAC289YY290C)**	61.182	46.941	87.089	65.07067	20.35453	0.012462
**sdhC(pRelAT298I)**	122.385	113.290	110.515	115.3967	6.209087	0.112277
**sdhC(pRelAQ264E)**	73.469	78.493	66.296	72.75267	6.129972	0.030187

Fig. S6a

SodA	Hfq			Hfq + SodA			Hfq + SodAΔSD		
	0'	30''	1'	0'	30''	1'	0'	30''	1'
Monomer	1026630	1184152	1259439	967850	1228442	1047480	1128352	1408590	1300903
Dimer	421740	1178032	899145	479570	908090	742255	493136	1144854	943281
Tetramer	10280	89000	87739	10070	80088	80440	9104	82438	83286

SodA

	Hfq			Hfq + SodA			Hfq + SodAΔSD		
	0'	30''	1'	0'	30''	1'	0'	30''	1'
Dimer	1	2.8	2.1	1	1.9	1.5	1	2.3	1.9
Tetramer	1	8.7	8.5	1	8.0	8.0	1	9.1	9.1

Fig. S6b

	Hfq + RelA			Hfq + SodA + RelA			Hfq + SodAΔSD + RelA		
	0'	30''	1'	0'	30''	1'	0'	30''	1'
Monomer	1111280	1245420	1298536	1134576	1373260	1547904	1082880	1213070	1391200
Dimer	722825	882376	964288	1245744	1847160	2281685	830631	915530	1111840
Tetramer	10610	89440	85504	13680	147480	165016	10551	85630	82360
Pentamer				1152	5460	6429			

	Hfq + RelA			Hfq + SodA + RelA			Hfq + SodAΔSD + RelA		
	0'	30''	1'	0'	30''	1'	0'	30''	1'
Dimer	1	1.2	1.3	1	1.5	1.8	1	1.1	1.3
Tetramer	1	8.4	8.1	1	10.8	12.1	1	8.1	7.8
Pentamer				1	4.7	5.6			

Fig. S6c

RyhB	Hfq			Hfq + RyhB			Hfq + RyhBm		
	0'	30''	1'	0'	30''	1'	0'	30''	1'
Monomer	1052424	693600	932466	862120	768220	823324	732010	961056	583917
Dimer	576408	1113600	1200600	456960	790088	1131164	273492	870048	489542
Tetramer	10144	64250	68440	12320	63684	65836	11882	67312	70128

	Hfq			Hfq + RyhB			Hfq + RyhBm		
	0'	30''	1'	0'	30''	1'	0'	30''	1'

Dimer	1	1.9	2.1	1	1.7	2.5	1	3.2	1.8
Tetramer	1	6.3	6.7	1	5.2	5.3	1	5.7	5.9

Fig. S6d

	Hfq + RelA			Hfq + RyhB + RelA			Hfq + RyhBm + RelA		
	0'	30''	1'	0'	30''	1'	0'	30''	1'
Monomer	543701	559054	557052	807424	820540	980784	720250	753522	745752
Dimer	210835	609180	560956	274664	717080	957888	229810	567798	653338
Tetramer	10278	69220	65534	12216	115820	219888	9,278	58366	60482
Pentamer				10792	68880	102960			

	Hfq + RelA			Hfq + RyhB + RelA			Hfq + RyhBm + RelA		
	0'	30''	1'	0'	30''	1'	0'	30''	1'
Dimer	1	2.9	2.7	1	2.6	3.5	1	2.5	2.8
Tetramer	1	6.7	6.4	1	9.5	18.0	1	6.3	6.5
Pentamer				1	6.4	9.5			

Fig. S7

b

	Hfq + RelA			Hfq + RyhB + RelA			Hfq + RyhBm + RelA		
	0'	30''	1'	0'	30''	1'	0'	30''	1'
Monomer	13830144	2537472	1238292	14317766	8515942	5551900	14544562	5233536	1185192
Dimer	8242816	5566296	4342400	7854906	21685214	22329022	8092086	7733012	5127100
Tetramer	484736	752840	788476	551886	3795824	4146874	671066	1506742	1371868
Pentamer				6136	100300	101952			

	Hfq + RelA			Hfq + RyhB + RelA			Hfq + RyhBm + RelA		
	0'	30''	1'	0'	30''	1'	0'	30''	1'
Dimer	1	0.7	0.5	1	2.8	2.8	1	1.0	0.6
Tetramer	1	1.6	1.6	1	6.9	7.5	1	2.2	2.0
Pentamer				1	16.3	16.6			

a

	Hfq + RelA			Hfq + SodA + RelA			Hfq + SodAm + RelA		
	0'	30''	1'	0'	30''	1'	0'	30''	1'
Monomer	18894078	725046	1770952	28426255	7461631	11947390	19956160	10460304	4046757
Dimer	6870024	2127680	4773738	8285277	26395934	32398135	8518440	10577664	8748454
Tetramer	352926	597190	1952610	732160	5049012	8918847	419720	1613088	1121406
Pentamer				745030	1326515	2155398			

	Hfq + RelA			Hfq + SodA + RelA			Hfq + SodAm + RelA		
	0'	30''	1'	0'	30''	1'	0'	30''	1'
Dimer	1	0.3	0.7	1	3.2	3.9	1	1.2	1.0
Tetramer	1	1.7	5.5	1	6.9	12.2	1	3.8	2.7
Pentamer				1	1.8	2.9			

Fig. S10b **Hfq:K56A**

	RyhB			SodA		
	0'	0.5'	5'	0'	0.5'	5'
Monomer	173686448	150326192	88520376	164321452	144503901	101640824
Dimer	21476222	11641864	25252352	36689492	311602938	467701280
Tetramer	1148780	531464	921692	694152	90936490	184902608
Pentamer				308016	20693302	57009432
Hexamer				796080	3883676	23524192

	RyhB		SodA	
	0.5'	5'	0.5'	5'
Dimer	0.5	1.2	8.5	12.7
Tetramer	0.5	0.8	131.0	266.4
Pentamer	0	0	67.2	185.1
Hexamer	0	0	4.9	29.6

Fig. S10b **Hfq:D9A**

	RyhB			SodA		
	0'	0.5'	1.5'	0'	0.5'	1.5'
Monomer	93052016	49726735	34913564	117991735	85022301	50349896
Dimer	52845928	68272235	79835136	113638405	547548882	421728864
Tetramer	2745840	7111960	9871038	6350195	64727481	65137020

	RyhB		SodA	
	0.5'	1.5'	0.5'	1.5'
Dimer	1.3	1.5	4.8	3.7
Tetramer	2.6	3.6	10.2	10.3

Fig. S10c **Hfq:G29A**

	RyhB			SodA		
	0'	0.5'	1.5'	0'	0.5'	1.5'
Monomer	91431528	65093652	54103512	92477055	41989541	20332466
Dimer	7973158	171507174	172016178	2325575	4261012	4976002
Tetramer	2298128	33230414	45412570	2083770	6915354	7675966

	RyhB		SodA	
	0.5'	1.5'	0.5'	1.5'

Dimer	21.5	21.6	1.8	2.1
Tetramer	14.5	19.8	3.3	3.7

Fig. S10d **Hfq:I30D**

	RyhB				SodA			
	0'	0.5'	1.5'	5'	0'	0.5'	1.5'	5'
Monomer	63470233	19588932	12729405	8022950	52033457	4998602	734648	89136
Dimer	8270132	70632012	63479695	68011190	1671943	7845748	4063096	1786608
Tetramer	1520702	14802348	19595155	26626730	262409	471030	445837	291600

	RyhB			SodA		
	0.5'	1.5'	5'	0.5'	1.5'	5'
Dimer	8.5	7.7	8.2	4.7	2.4	1.1
Tetramer	9.7	12.9	17.5	1.8	1.7	1.1

Fig. S12a

	RyhB			SodA		
	0'	0.5'	5'	0'	0.5'	5'
Monomer	1018003	856152	801500	828208	856548	590224
Dimer	146349	222075	382760	455988	1161314	970584
Tetramer	15939	18330	57720	12580	497882	510896
Pentamer				9768	106414	167388

	RyhB			SodA		
	0'	0.5'	5'	0'	0.5'	5'
Dimer	1	1.5	2.6	1	2.5	2.1
Tetramer	1	1.2	3.6	1	39.6	40.6
Pentamer				1	10.9	17.1

Fig. S12b

	RyhB			SodA		
	0'	0.5'	1.5'	0'	0.5'	1.5'
Monomer	86027850	39354675	41468469	1.58E+08	1.28E+08	1.6E+08
Dimer	39855150	40958007	40473895	1.08E+08	1.91E+08	2.3E+08
Tetramer	6951750	11473647	5913978	6453156	42910443	5.4E+07

	RyhB			SodA		
	0'	0.5'	1.5'	0'	0.5'	1.5'
Dimer	1	1.0	1.0	1	1.8	2.1
Tetramer	1	1.7	0.9	1	6.6	8.4

Fig. S12c

	RyhB			SodA		
	0'	0.5'	1.5'	0'	0.5'	1.5'
Monomer	932217	726596	468017	826919	486135	321935
Dimer	326040	573835	655946	197820	222615	237150
Tetramer	15015	124030	185888	1785	2095	4440
Pentamer	1320	2710	4427			

	RyhB			SodA		
	0'	0.5'	1.5'	0'	0.5'	1.5'
Dimer	1	1.8	2.0	1	1.1	1.2
Tetramer	1	8.3	12.4	1	1.2	2.5
Pentamer	1	2.1	3.4			

Fig. S12d

	RyhB			SodA		
	0'	0.5'	5'	0'	0.5'	5'
Monomer	618772	432144	360220	708864	384624	337295
Dimer	181056	155232	235600	105648	78336	190946
Tetramer	656	4320	8560	994	864	6601

	RyhB			SodA		
	0'	0.5'	5'	0'	0.5'	5'
Dimer	1	0.9	1.3	1	0.7	1.8
Tetramer	1	6.6	13.0	1	0.9	6.6

Fig. S14c

RyhB	Total copies (X 10^6)	16S	Total copies (X 10^2)	Total copies (X 10^6)
pEF (1:1000000) (1)	501	pEF (1:100) (1)	68,235	7
pEF (1:1000000) (1)	523	pEF (1:100) (1)	73,894	7.3894
pEF (1:1000000) (2)	504	pEF (1:100) (2)	61,232	6
pEF (1:1000000) (2)	507	pEF (1:100) (2)	54,408	5.4408
pEF (1:1000000) (3)	506	pEF (1:100) (3)	53,299	5
pEF (1:1000000) (3)	503	pEF (1:100) (3)	59,799	6
pEF (1:1000000) (1)	555	pEF (1:100) (1)	54,893	6
pEF (1:1000000) (1)	559	pEF (1:100) (1)	92,813	9.3
pEF (1:1000000) (2)	558	pEF (1:100) (2)	47,764	5
pEF (1:1000000) (2)	558	pEF (1:100) (2)	111,715	11.2
pEF (1:1000000) (3)	575	pEF (1:100) (3)	58,506	6
pEF (1:1000000) (3)	560	pEF (1:100) (3)	59,851	6
RelA (1:1000000) (1)	869	RelA (1:100) (1)	58,798	6
RelA (1:1000000) (1)	865	RelA (1:100) (1)	57,199	5.7199
RelA (1:1000000) (2)	729	RelA (1:100) (2)	59,272	6
RelA (1:1000000) (2)	768	RelA (1:100) (2)	63,270	6.327
RelA (1:1000000) (3)	1,161	RelA (1:100) (3)	70,158	7
RelA (1:1000000) (3)	1,005	RelA (1:100) (3)	68,081	6.8081
ΔNTD (1:1000000) (1)	490	ΔNTD (1:100) (1)	61,131	6
ΔNTD (1:1000000) (1)	498	ΔNTD (1:100) (1)	67,550	6.755
ΔNTD (1:1000000) (2)	495	ΔNTD (1:100) (2)	70,651	7
ΔNTD (1:1000000) (2)	481	ΔNTD (1:100) (2)	70,274	7.0274
ΔNTD (1:1000000) (3)	490	ΔNTD (1:100) (3)	67,634	7
ΔNTD (1:1000000) (3)	493	ΔNTD (1:100) (3)	68,495	6.8495
ΔCTD (1:1000000) (1)	1,029	ΔCTD (1:100) (1)	71,330	7
ΔCTD (1:1000000) (1)	880	ΔCTD (1:100) (1)	72,403	7.2403
ΔCTD (1:1000000) (2)	872	ΔCTD (1:100) (2)	59,886	6
ΔCTD (1:1000000) (2)	988	ΔCTD (1:100) (2)	64,939	6.4939
ΔCTD (1:1000000) (3)	1,067	ΔCTD (1:100) (3)	75,571	8
ΔCTD (1:1000000) (3)	987	ΔCTD (1:100) (3)	75,400	7.54
C289Y (1:1000000) (1)	573	C289Y (1:100) (1)	62,708	6
C289Y (1:1000000) (1)	573	C289Y (1:100) (1)	65,740	6.6
C289Y (1:1000000) (2)	571	C289Y (1:100) (2)	74,894	8
C289Y (1:1000000) (2)	573	C289Y (1:100) (2)	71,025	7.1
C289Y (1:1000000) (3)	573	C289Y (1:100) (3)	66,912	7
C289Y (1:1000000) (3)	569	C289Y (1:100) (3)	71,454	7.1
C289A (1:1000000) (1)	1,047	C289A (1:100) (1)	66,381	7
C289A (1:1000000) (1)	1,015	C289A (1:100) (1)	65,358	6.5
C289A (1:1000000) (2)	975	C289A (1:100) (2)	62,259	6
C289A (1:1000000) (2)	956	C289A (1:100) (2)	57,782	5.7
C289A (1:1000000) (3)	883	C289A (1:100) (3)	83,487	8
C289A (1:1000000) (3)	782	C289A (1:100) (3)	79,374	8
CYYC (1:1000000) (1)	718	CYYC (1:100) (1)	75,186	8
CYYC (1:1000000) (1)	820	CYYC (1:100) (1)	83,131	8.3
CYYC (1:1000000) (2)	1,060	CYYC (1:100) (2)	74,691	8

CYYC (1:1000000) (2)	1,006	CYYC (1:100) (2)	57,502	5.7
CYYC (1:1000000) (3)	700	CYYC (1:100) (3)	65,650	7
CYYC (1:1000000) (3)	700	CYYC (1:100) (3)	56,014	5.6
T298I (1:1000000) (1)	526	T298I (1:100) (1)	84,713	9
T298I (1:1000000) (1)	438	T298I (1:100) (1)	89,537	8.9
T298I (1:1000000) (2)	448	T298I (1:100) (2)	83,796	8
T298I (1:1000000) (2)	479	T298I (1:100) (2)	80,070	8
T298I (1:1000000) (3)	550	T298I (1:100) (3)	75,457	8
T298I (1:1000000) (3)	540	T298I (1:100) (3)	83,301	8.3
Q264E (1:1000000) (1)	877	Q264E (1:100) (1)	67,880	7
Q264E (1:1000000) (1)	1,070	Q264E (1:100) (1)	76,964	7.7
Q264E (1:1000000) (2)	829	Q264E (1:100) (2)	86,149	9
Q264E (1:1000000) (2)	887	Q264E (1:100) (2)	97,101	9.7
Q264E (1:1000000) (3)	845	Q264E (1:100) (3)	91,055	9
Q264E (1:1000000) (3)	748	Q264E (1:100) (3)	89,184	8.9

Fig. S17

RyhB copy no.

Strains	Before Co-IP (Total RNA)	After Co-IP
relA	42,666	9,537
	44,785	9,966
	44,839	10,404
	44,147	9,767
	34,169	8,486
	44,297	7,476
relA C289Y	39,201	0.43340
	39,943	0.42285
	40,725	0.41355
	38,951	0.44550
	36,498	0.03840
	37,559	0.39475
relA Δhfq	13,278	5,473
	13,464	5,922
	12,234	5,506
	11,297	5,360
	12,602	5,525
	13,027	5,693
rel C289Y Δhfq	11,687	0.37655
	13,577	0.42080
	8,115	0.47100
	8,739	0.46465
	10,571	0.45775
	10,567	0.47665

McaS copy no.

Strains	Before Co-IP (Total RNA)	After Co-IP
relA	466,788	25000
	471,954	19000
	412,338	31000
	433,750	26000
	411,205	25000
	421,746	28000
relA C289Y	301,357	29
	319,457	30
	329,093	35
	328,008	30
	336,750	33
	343,110	35
relA Δhfq	242,591	9000

	254,627	11000
	267,761	14000
	275,594	12000
	225,694	13000
	204,208	13000
rel C289Y Δhfq	331,857	32
	379,628	35
	281,201	31
	243,580	32
	257,870	26
	245,032	25

SraC copy no.

Strains	Before Co-IP (Total RNA)	After Co-IP
relA	101,107	8170
	96,532	10180
	97,341	9040
	96,936	8880
	97,275	11090
	97,843	10750
relA C289Y	84,815	107
	78,515	103
	47,990	111
	83,946	107
	92,684	109
	67,272	106
relA Δhfq	93,552	11360
	95,063	11520
	95,832	12700
	85,758	12030
	94,172	13120
	99,481	12340
rel C289Y Δhfq	102,650	111
	91,949	109
	96,382	123
	84,379	120
	89,223	109
	84,864	111

MgrR copy no.

Strains	Before Co-IP (Total RNA)	After Co-IP
relA	384200	27040
	422300	24930
	363400	21890
	439900	26800
	453900	22970
	430700	23020
relA C289Y	368000	24230
	350400	22620
	283700	22070
	385500	27070
	412100	21510
	349600	21880
relA Δhfq	183600	21680
	278600	26910
	291500	19540
	221300	21990
	201600	19570
	261500	19900
rel C289Y Δhfq	149300	20440
	151000	23510
	112900	23120
	102400	21000
	112000	21220
	101900	23250

MicC copy no.

Strains	Before Co-IP (Total RNA)	After Co-IP
relA	12,160	90
	13,574	86
	10,783	83
	11,205	86
	10,195	106
	9,761	92
relA C289Y	10,803	119
	10,554	128
	10,338	114
	9,007	114
	9,494	100
	9,332	120
relA Δhfq	5,306	89
	5,435	96

	5,391	146
	5,469	192
	4,794	148
	6,026	128
rel C289Y Δhfq	4,960	122
	5,427	122
	5,954	106
	5,913	110
	5,648	119
	6,283	115

sodA **copy no.**

Strains	Before Co-IP (Total RNA)	After Co-IP
relA	218000	20820
	210000	21820
	210000	16510
	226200	23340
	172100	18220
	160700	20950
relA C289Y	565000	12340
	580100	11410
	539100	10640
	515100	13770
	452800	13950
	626400	13110
relA Δhfq	392800	17490
	398400	21490
	704400	13210
	544800	16870
	446600	15150
	526800	15700
rel C289Y Δhfq	476900	12740
	645300	15630
	377100	11680
	432400	11100
	420600	11300
	391700	11500

***sdhC* copy no.**

Strains	Before Co-IP (Total RNA)	After Co-IP
relA	197800	50300
	175200	44680
	186200	39060
	209500	46560
	211800	49480
	204500	39020
relA C289Y	464800	16310
	463700	17840
	453400	18560
	474900	18980
	485700	17540
	548600	21020
relA Δhfq	396700	44770
	406100	48190
	403600	46510
	414400	45330
	467200	50340
		50560
rel C289Y Δhfq	461000	16140
	504500	17870
	385800	18700
	386300	19610
	381500	19080
	386100	21930

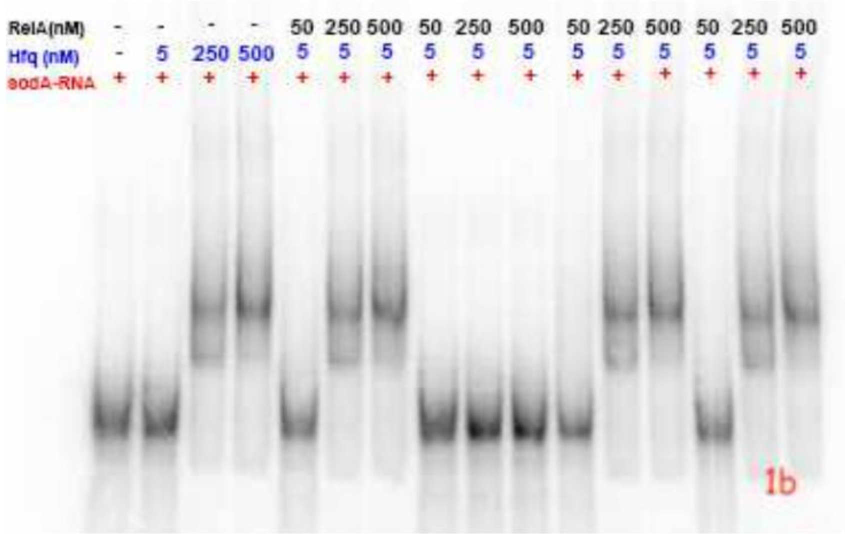

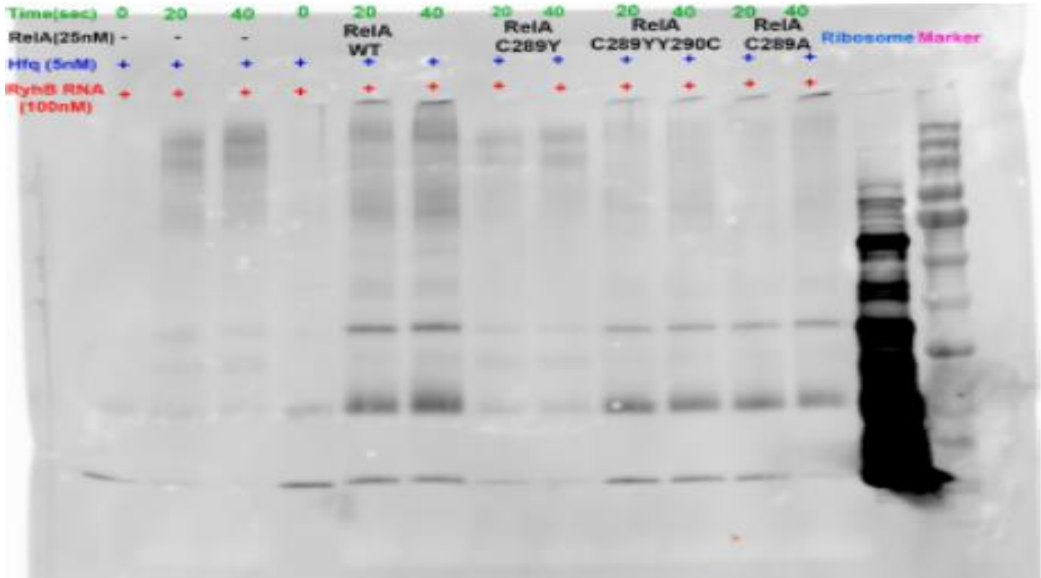

1c
Anti-Hfq antibody

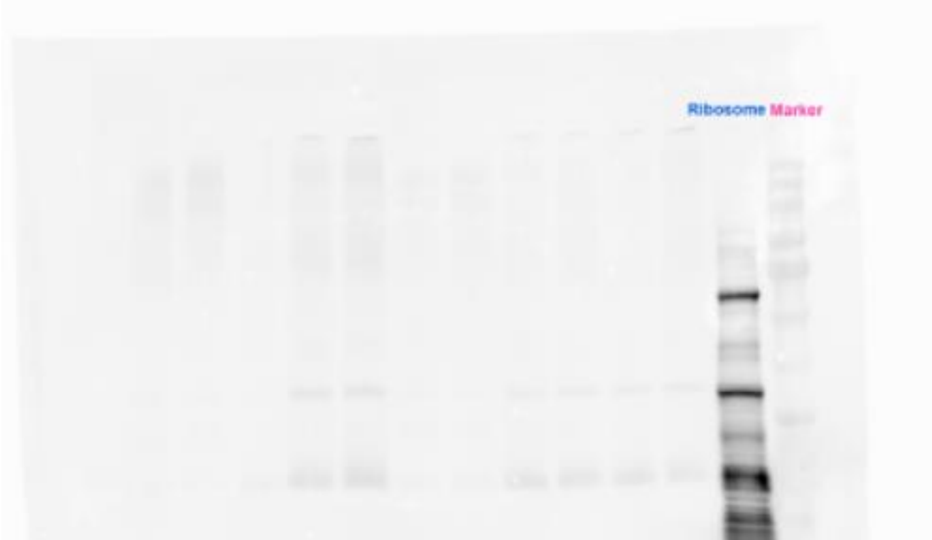

1c low exposure same gel to clearly see the ribosome bands
Anti-Hfq antibody

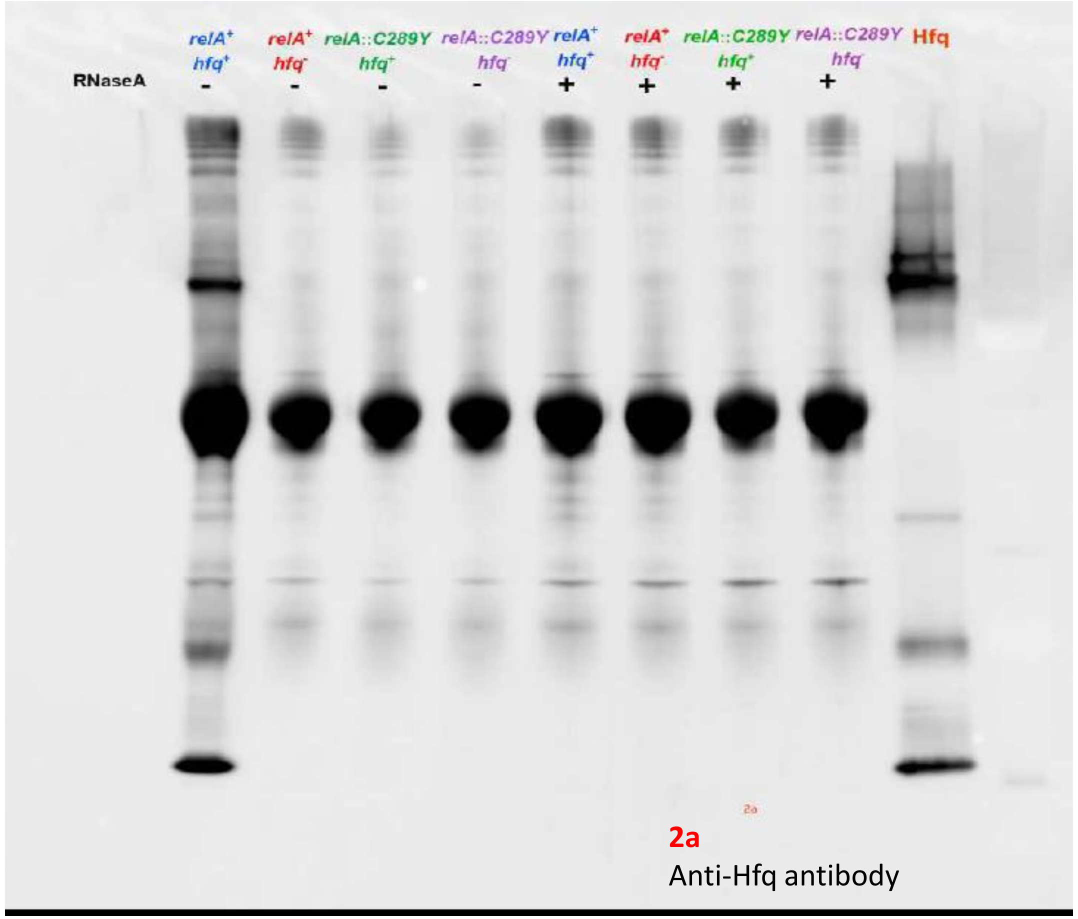
relA+ hfq+
relA+ hfq-
relA::C289Y hfq+
relA::C289Y hfq-
relA+ hfq+
relA+ hfq-
relA::C289Y hfq+
relA::C289Y hfq-
Hfq
RNaseA
- - - - + + + +
2a
Anti-Hfq antibody

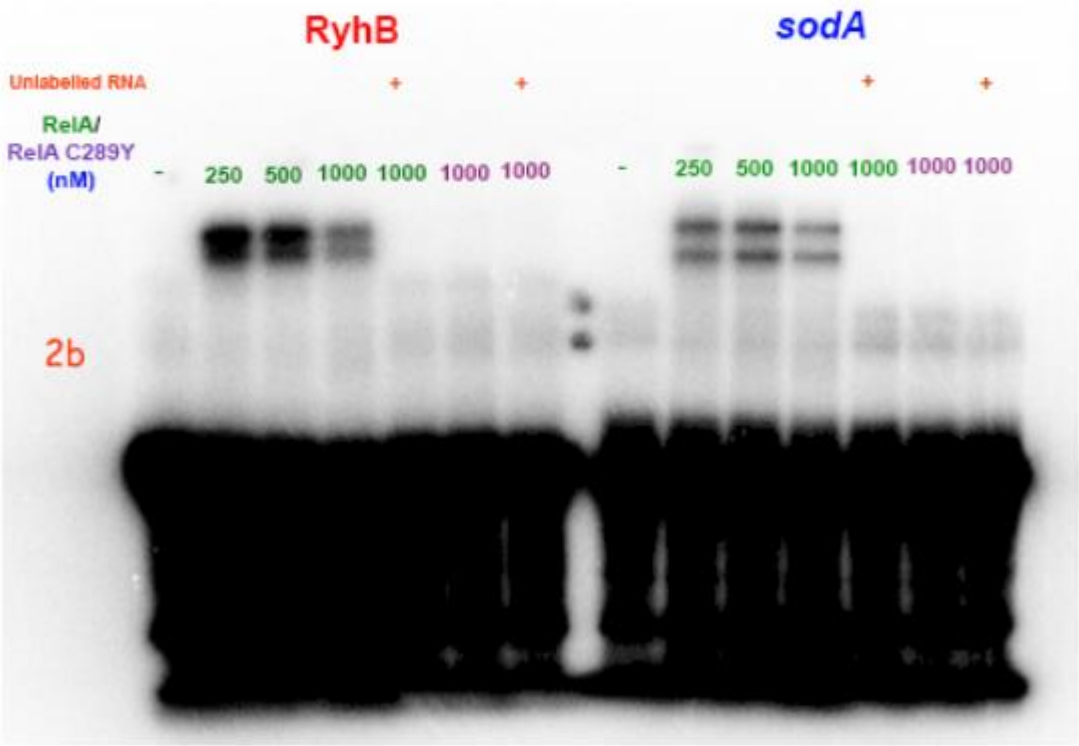
RyhB
sodA
Unlabelled RNA
+ + + +
RelA/
RelA C289Y
(nM)
- 250 500 1000 1000 1000 1000
- 250 500 1000 1000 1000 1000
2b

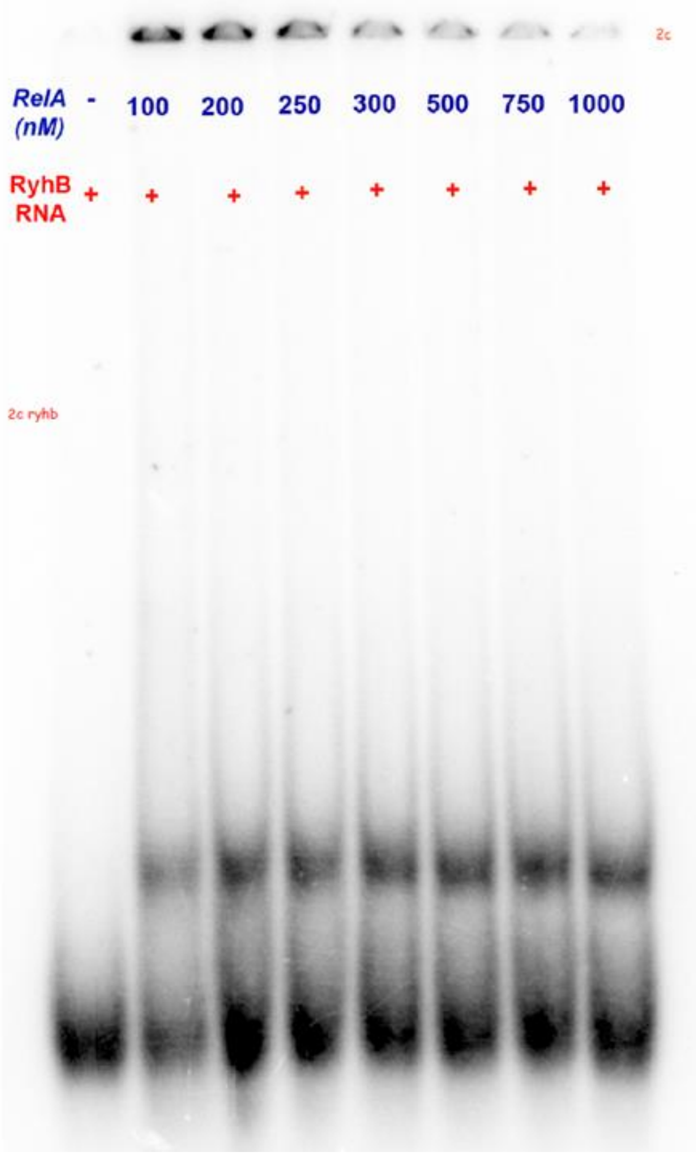
2c
RelA (nM)
-
100
200
250
300
500
750
1000
RyhB RNA
+
+
+
+
+
+
+
+
2c ryhb

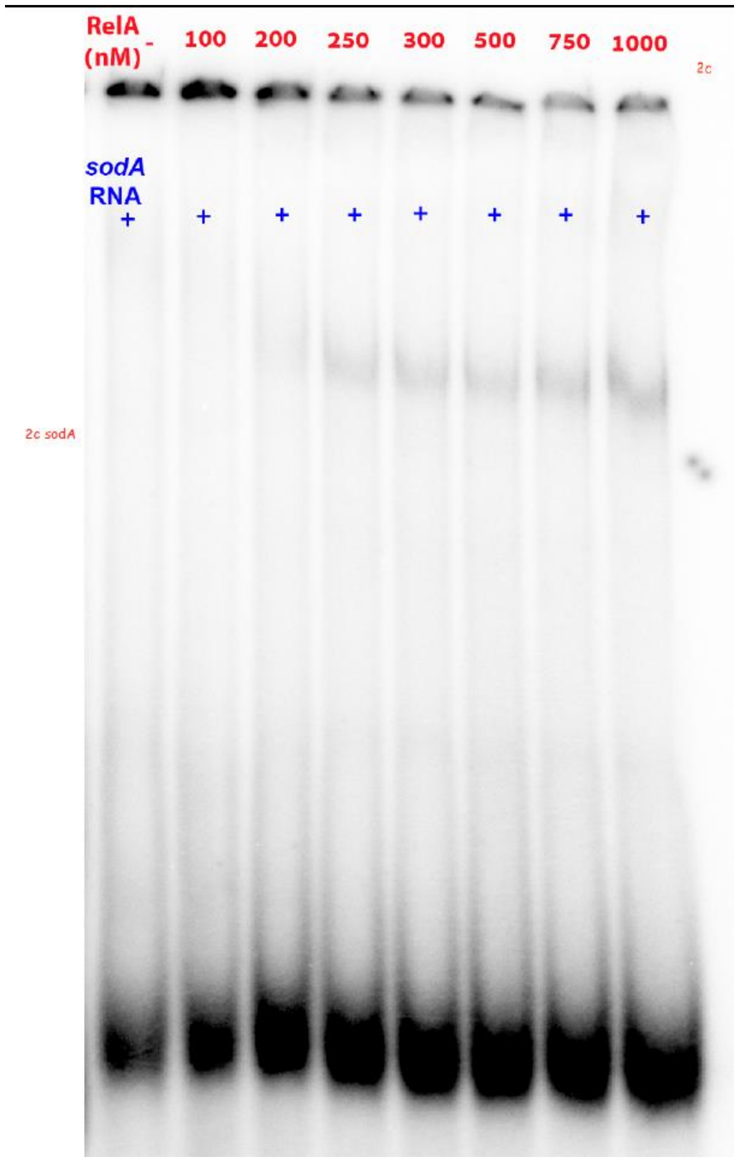
RelA (nM)
-
100
200
250
300
500
750
1000
2c
sodA RNA
+
+
+
+
+
+
+
+
2c sodA

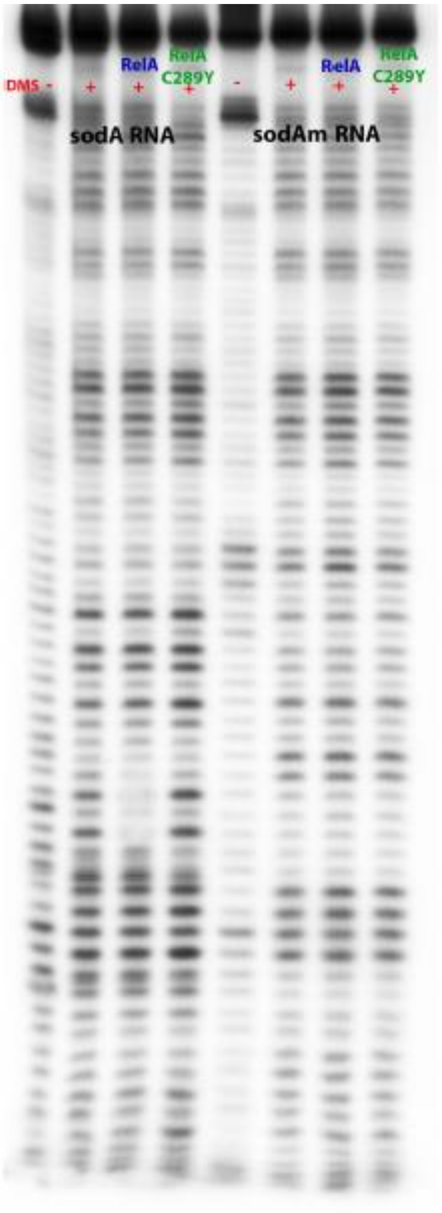

3a

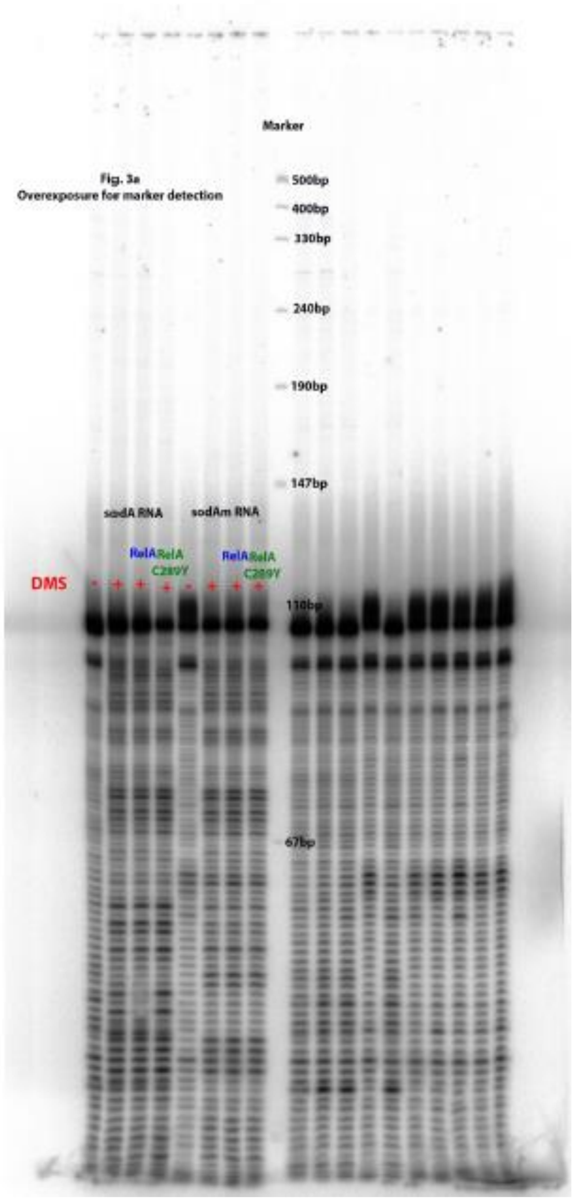

3a same gel high exposure to see marker bands

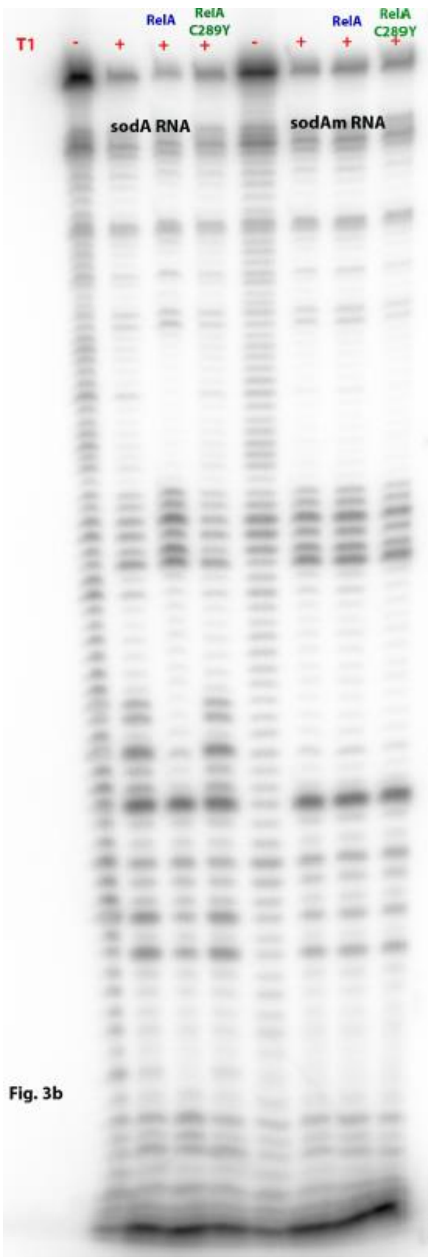

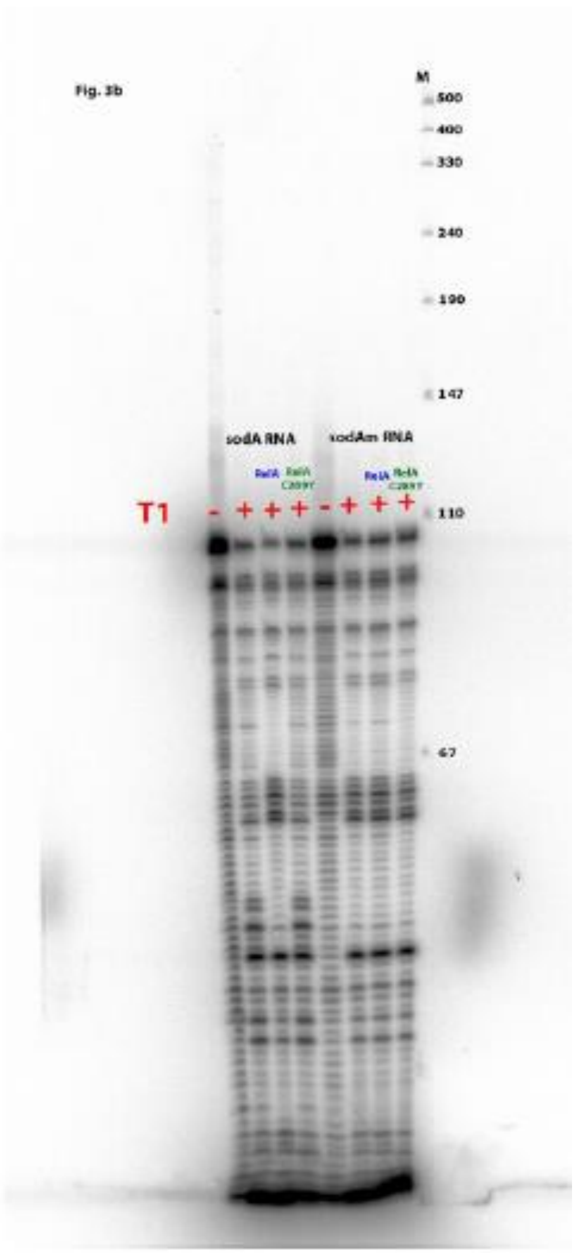

3b same gel high exposure to see marker bands

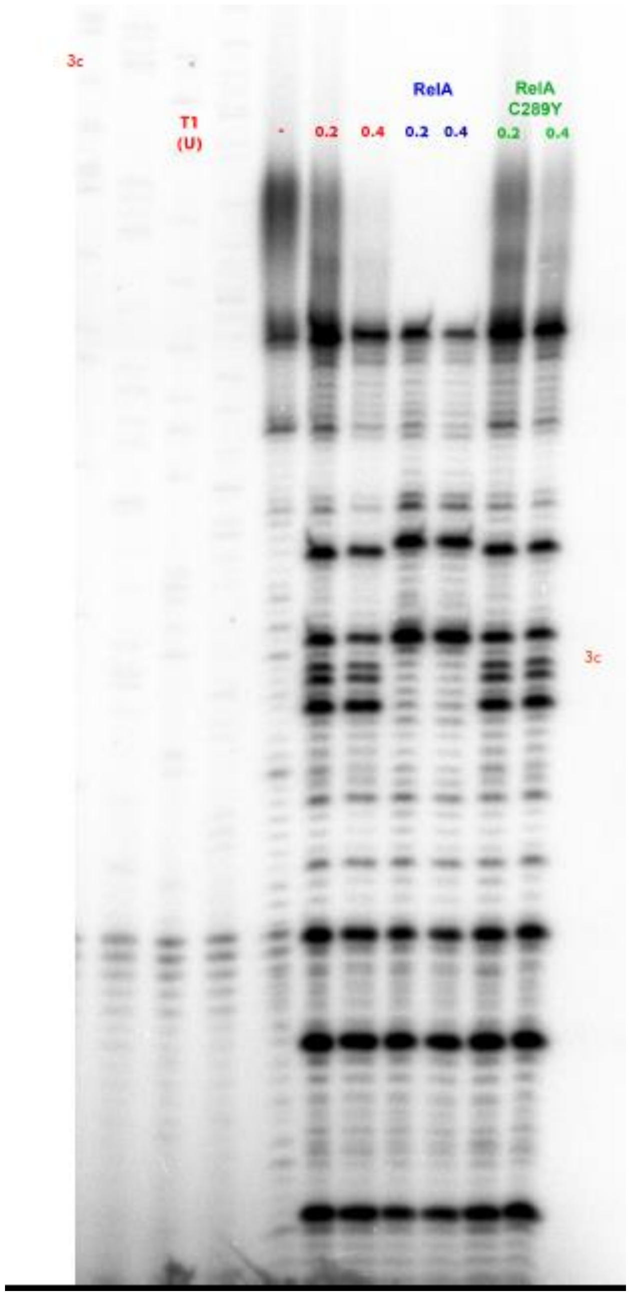

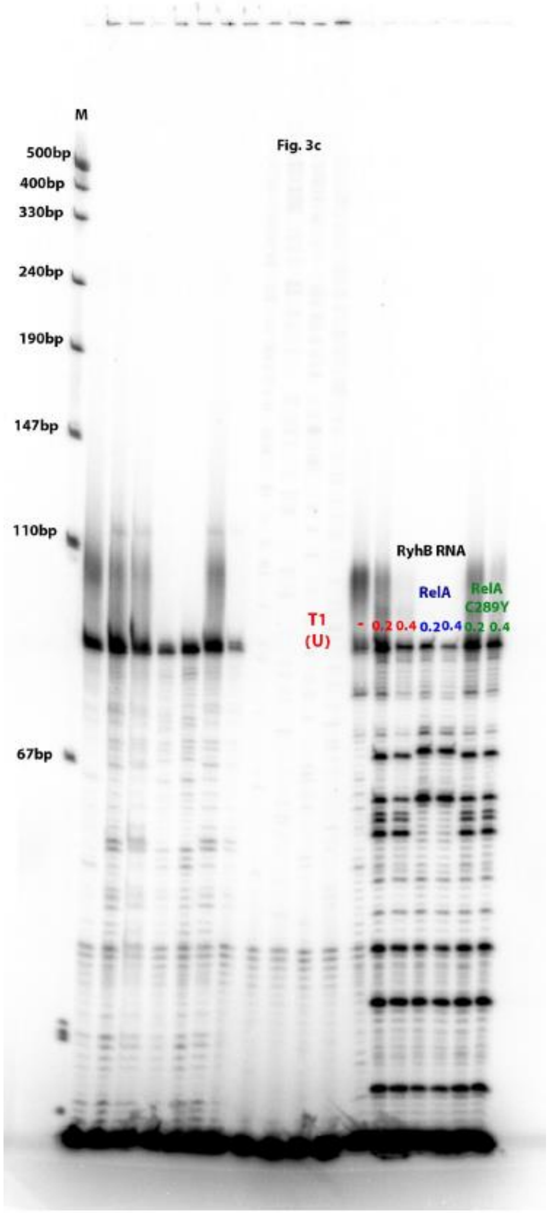

3c same gel high exposure to see marker bands

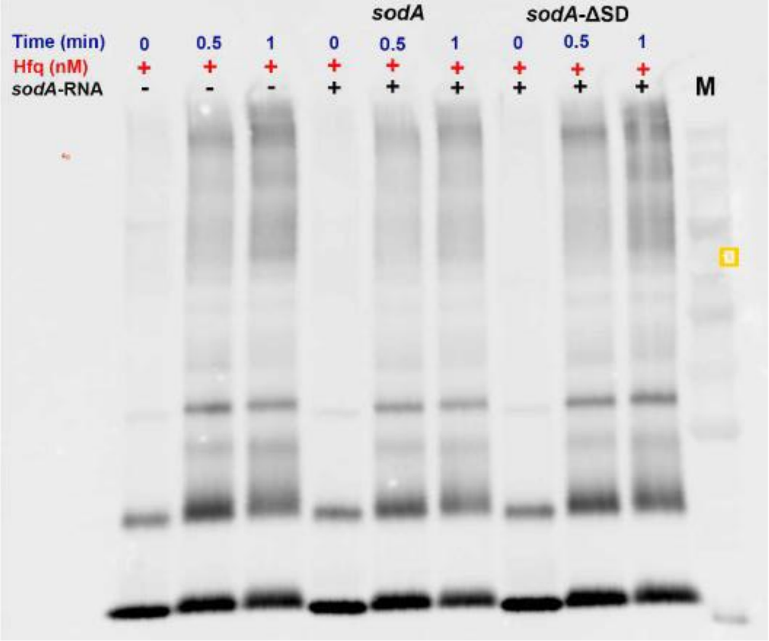

4a
Anti-Hfq antibody

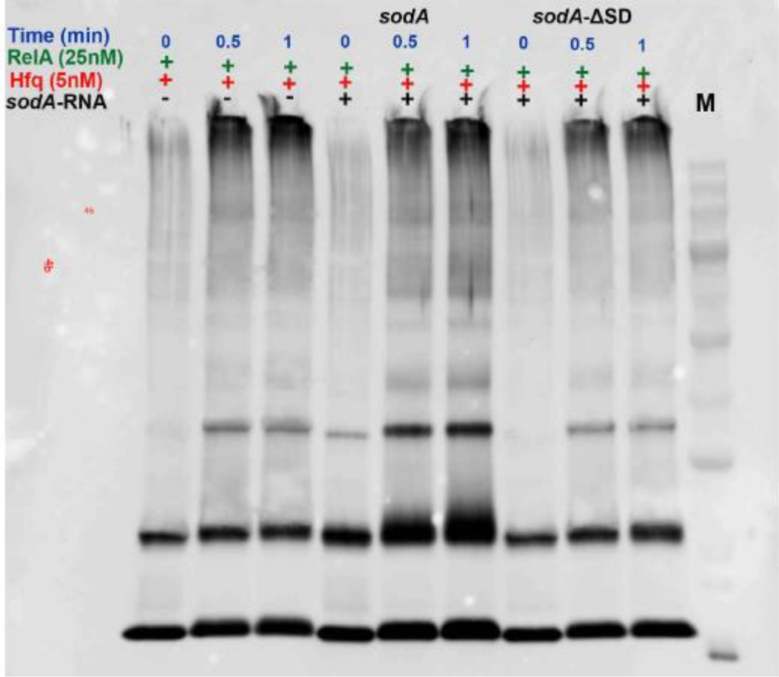

4b
Anti-Hfq antibody

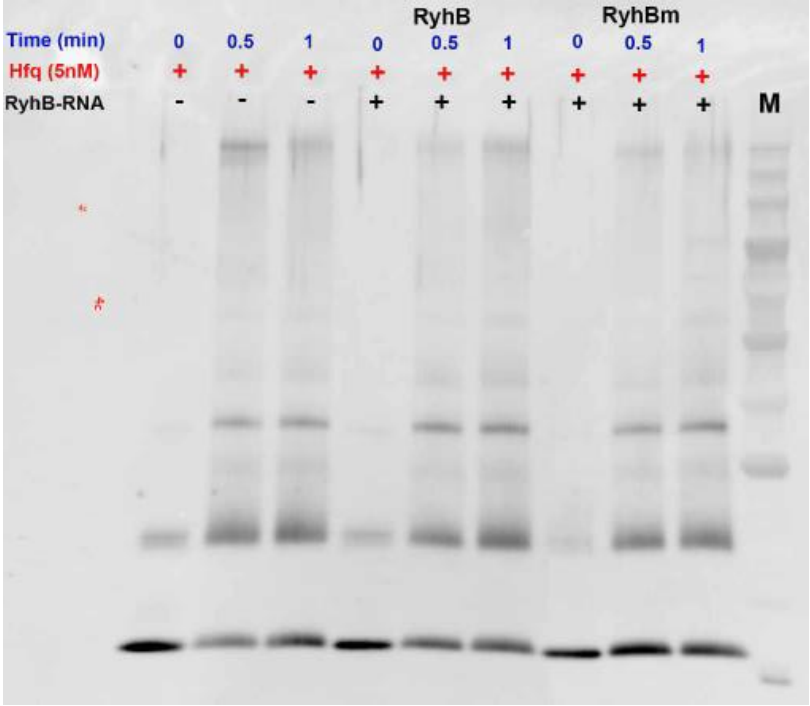

4c
Anti-Hfq antibody

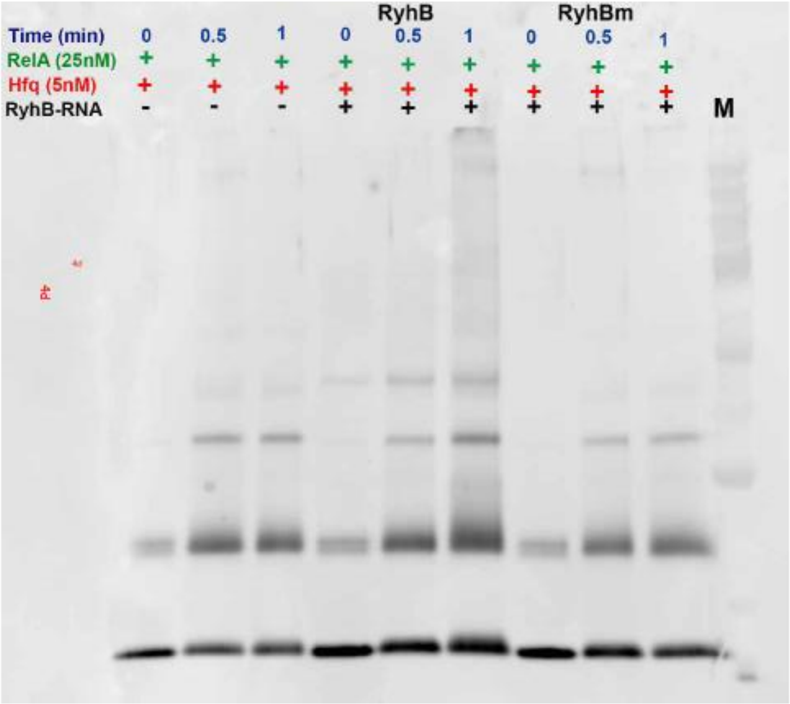

4d
Anti-Hfq antibody

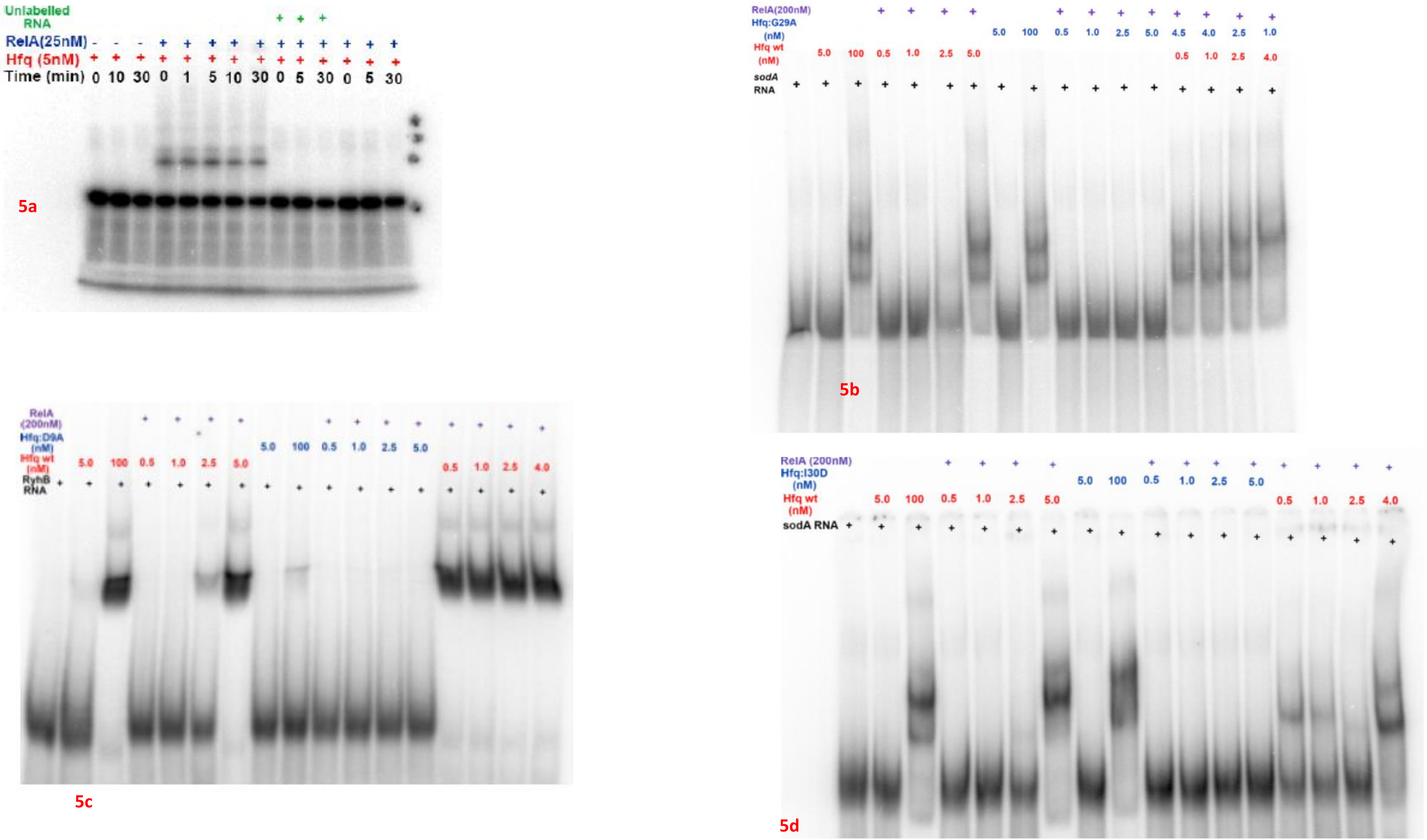
Unlabelled RNA + + +
RelA(25nM) - - - + + + + + + + + + + +
Hfq (5nM) + + + + + + + + + + + + + +
Time (min) 0 10 30 0 1 5 10 30 0 5 30 0 5 30
5a
RelA(200nM)
Hfq:G29A (nM) 5.0 100 0.5 1.0 2.5 5.0 4.5 4.0 2.5 1.0
Hfq wt (nM) 5.0 100 0.5 1.0 2.5 5.0 0.5 1.0 2.5 4.0
sodA RNA
5b
RelA (200nM)
Hfq:D9A (nM) 5.0 100 0.5 1.0 2.5 5.0
Hfq wt (nM) 5.0 100 0.5 1.0 2.5 5.0 0.5 1.0 2.5 4.0
RyhB RNA
5c
RelA (200nM)
Hfq:I30D (nM) 5.0 100 0.5 1.0 2.5 5.0
Hfq wt (nM) 5.0 100 0.5 1.0 2.5 5.0 0.5 1.0 2.5 4.0
sodA RNA
5d

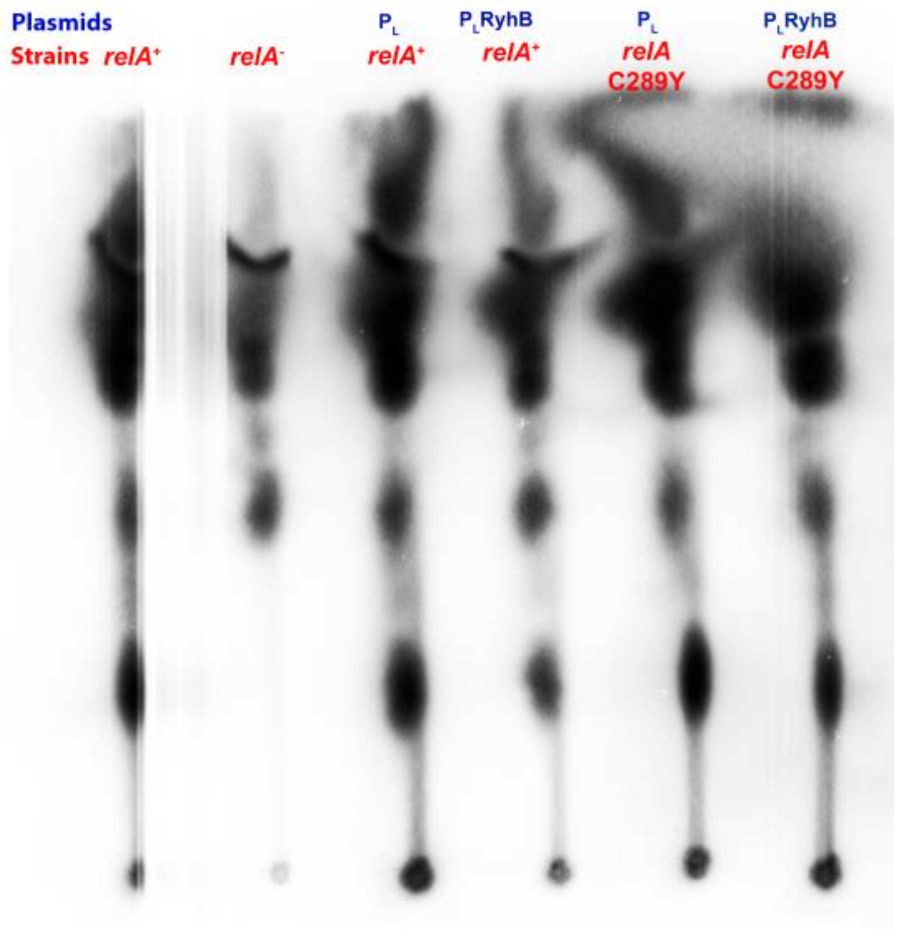

Plasmids
Strains relA+
relA-
P_L
relA+
P_L RyhB
relA+
P_L
relA
C289Y
P_L RyhB
relA
C289Y

6a

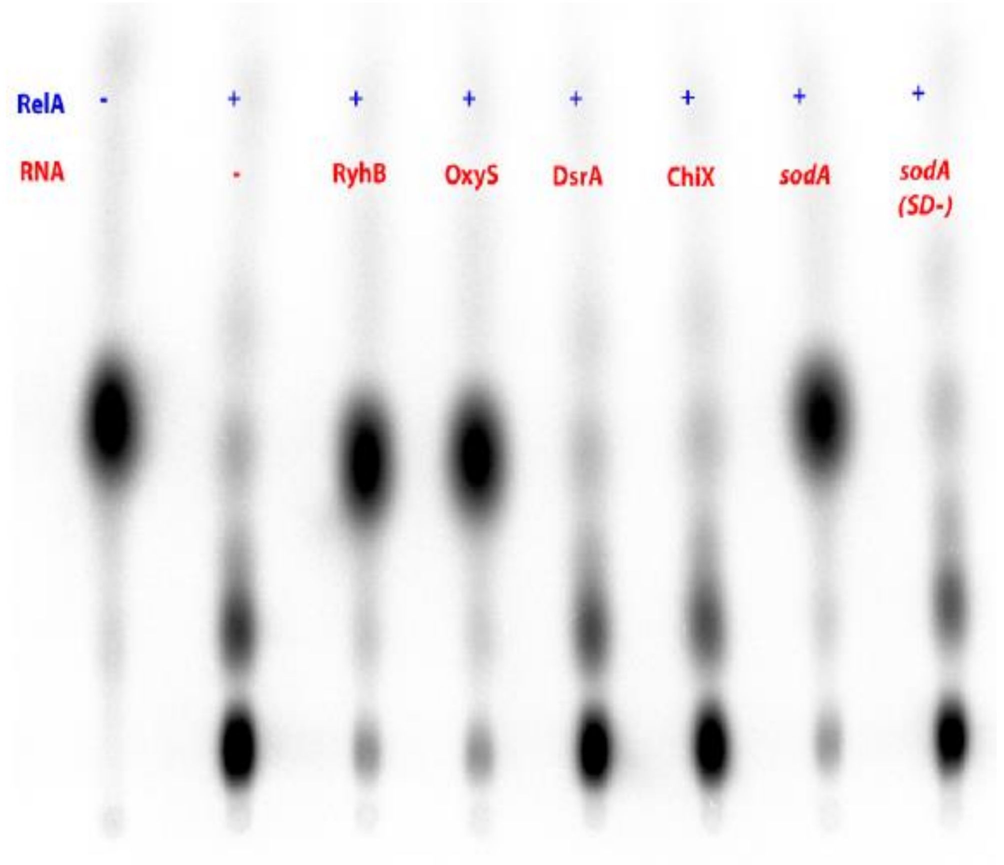

RelA - + + + + + + +
RNA - RyhB OxyS DsrA ChiX sodA sodA (SD-)

6b

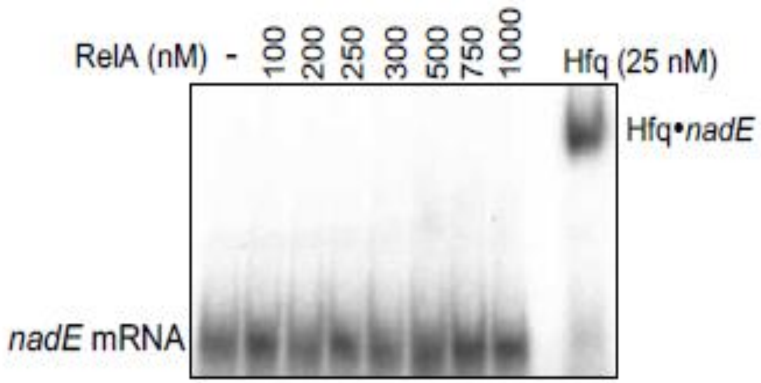

Gel mobility shift assay of radiolabeled *nadE* mRNA (210 nt) incubated with increasing concentrations of RelA as indicated. Incubations were carried out at 22°C for 10 min. The products were UV cross-linked before loading on 4% native gel electrophoresis.

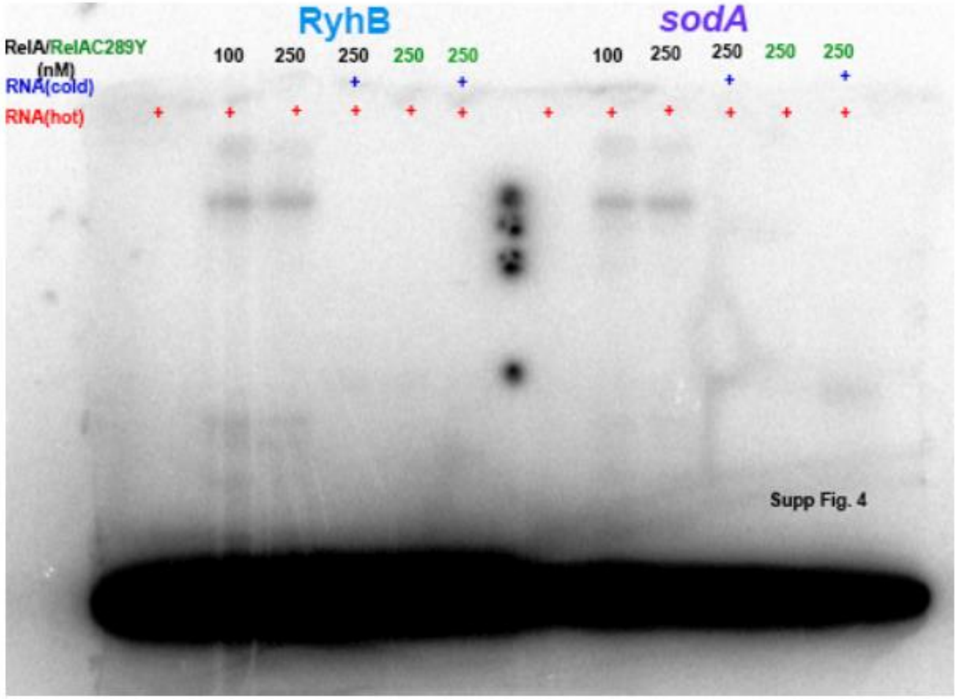
RyhB
sodA
RelA/RelAC289Y
(nM)
RNA(cold)
RNA(hot)
100 250 250 250 250
100 250 250 250 250
Supp Fig. 4

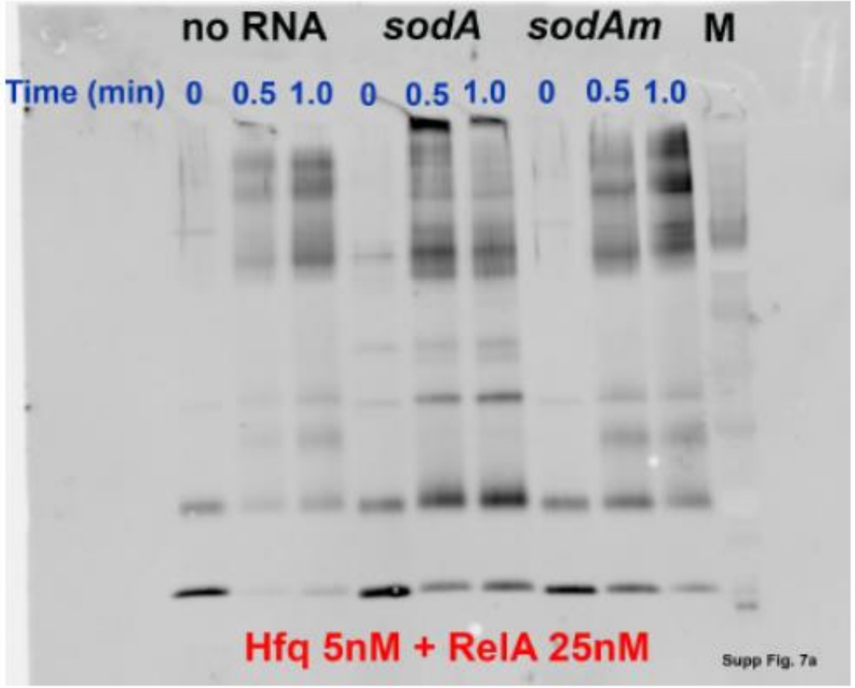

Anti-Hfq antibody

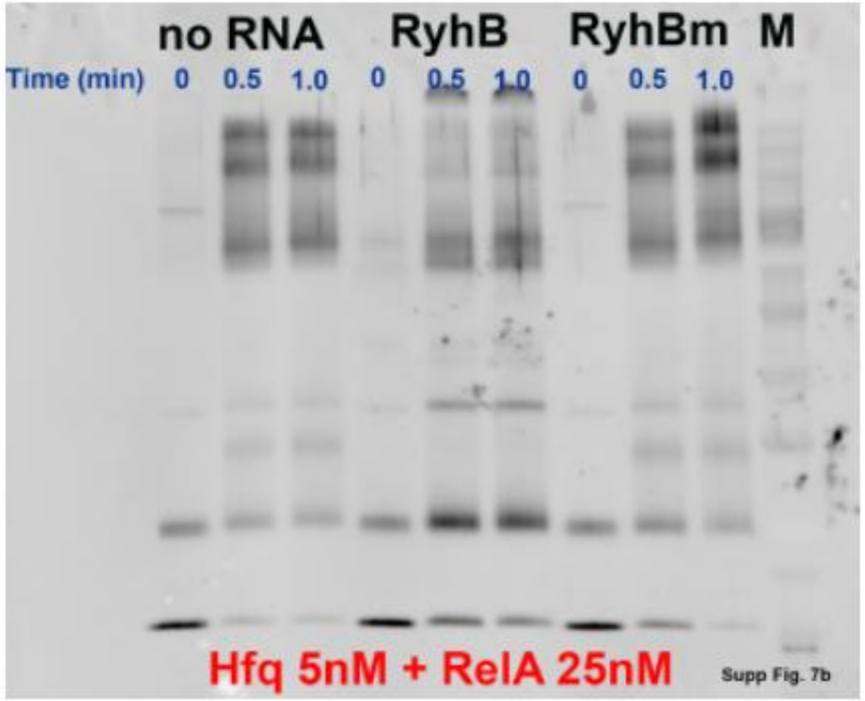

Anti-Hfq antibody

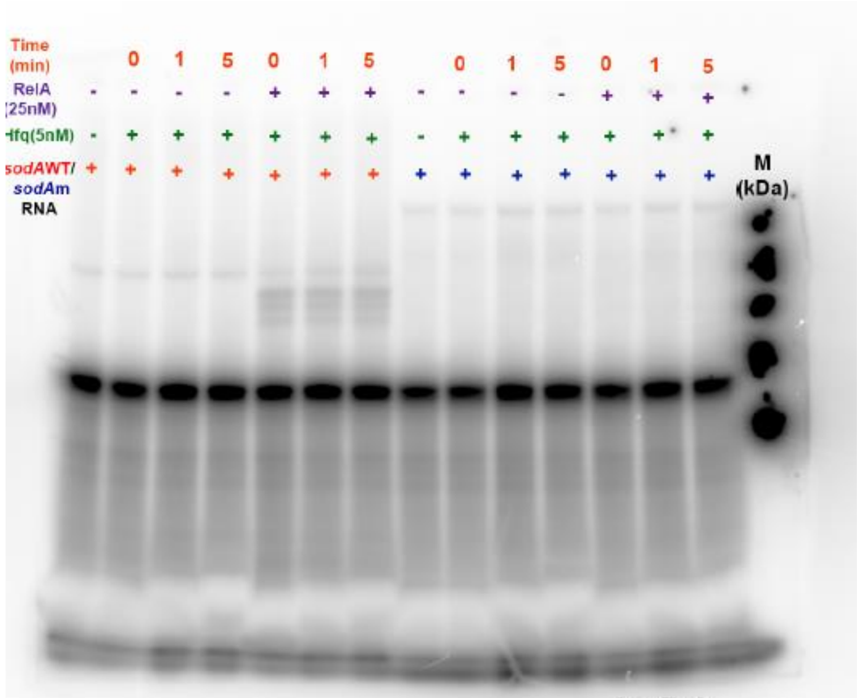
Time (min)
0 1 5 0 1 5 0 1 5 0 1 5
RelA (25nM)
Hfq(5nM)
sodAWT/ sodAm RNA
M (kDa)

Supp Fig. 8

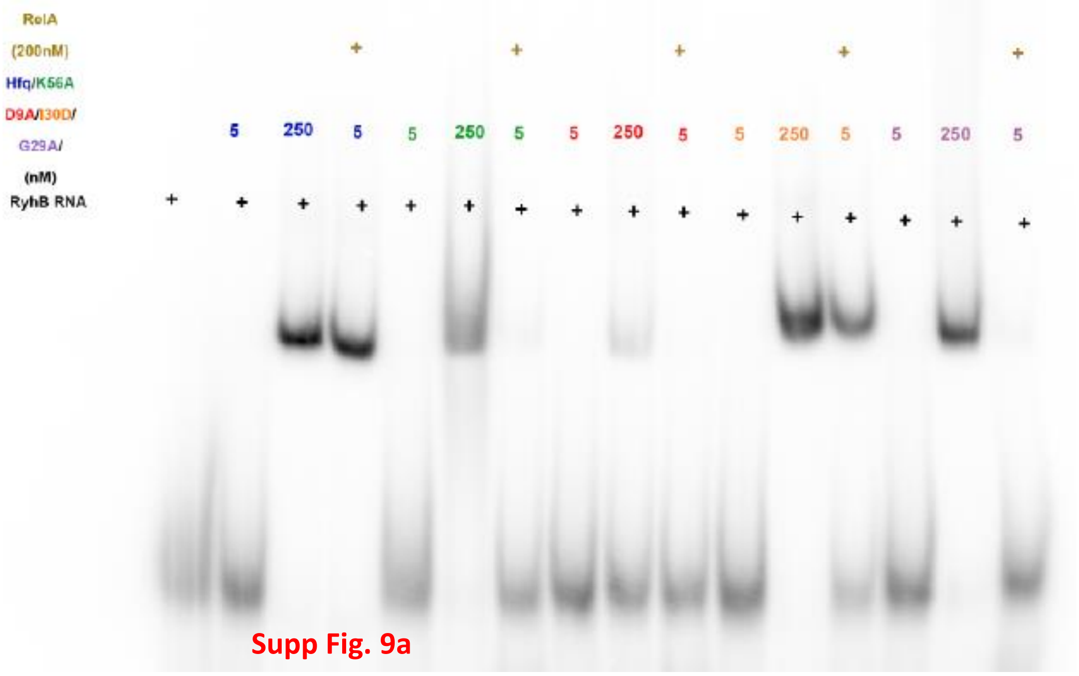

Supp Fig. 9a

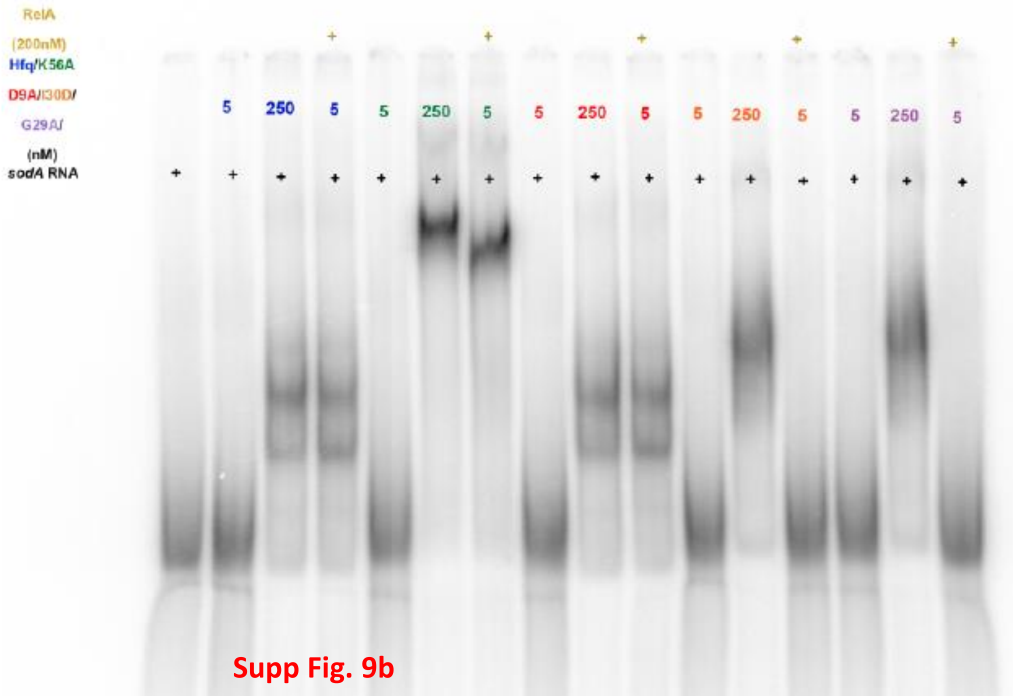

Supp Fig. 9b

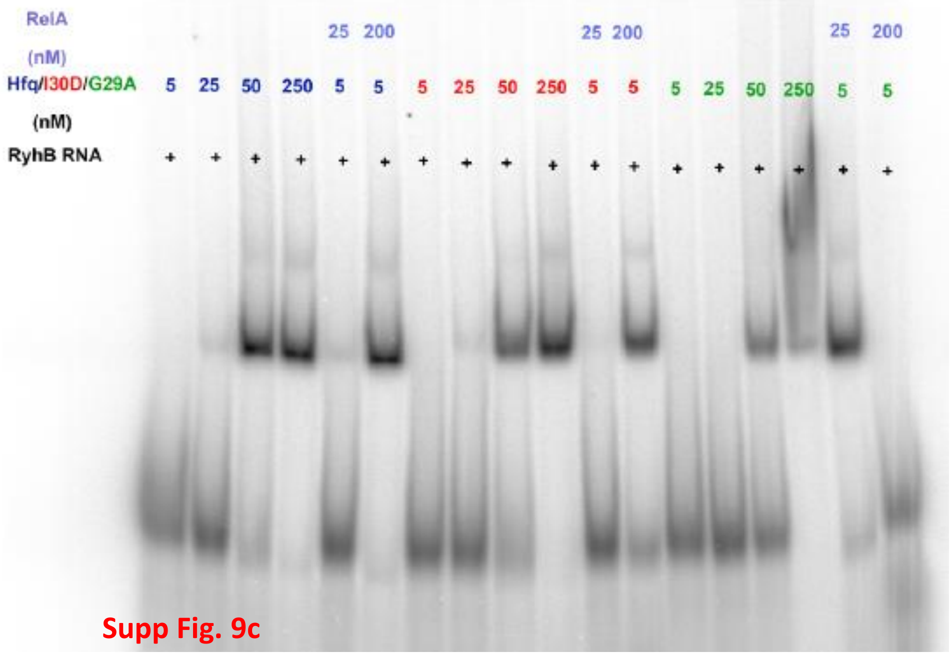

Supp Fig. 9c

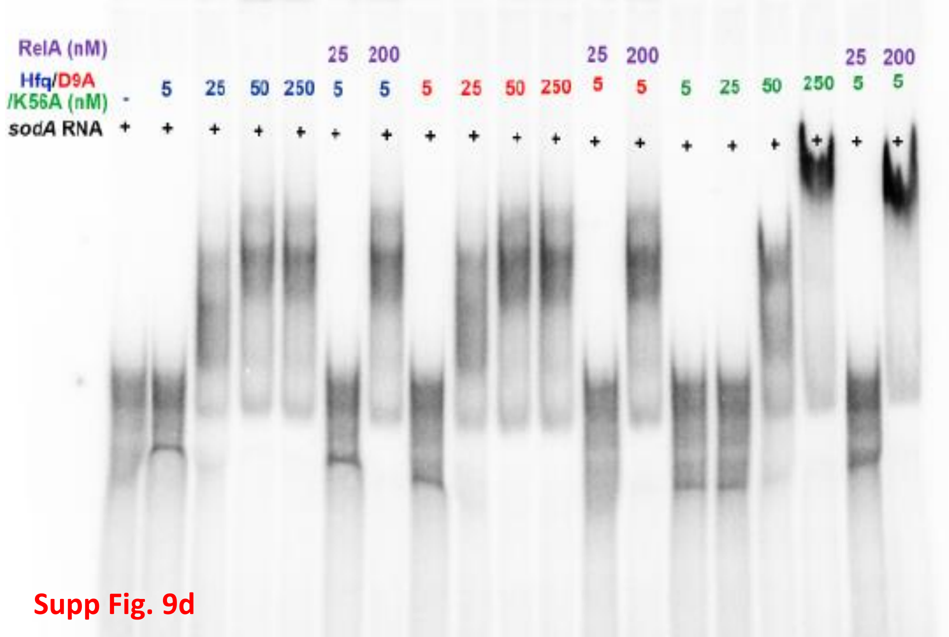

Supp Fig. 9d

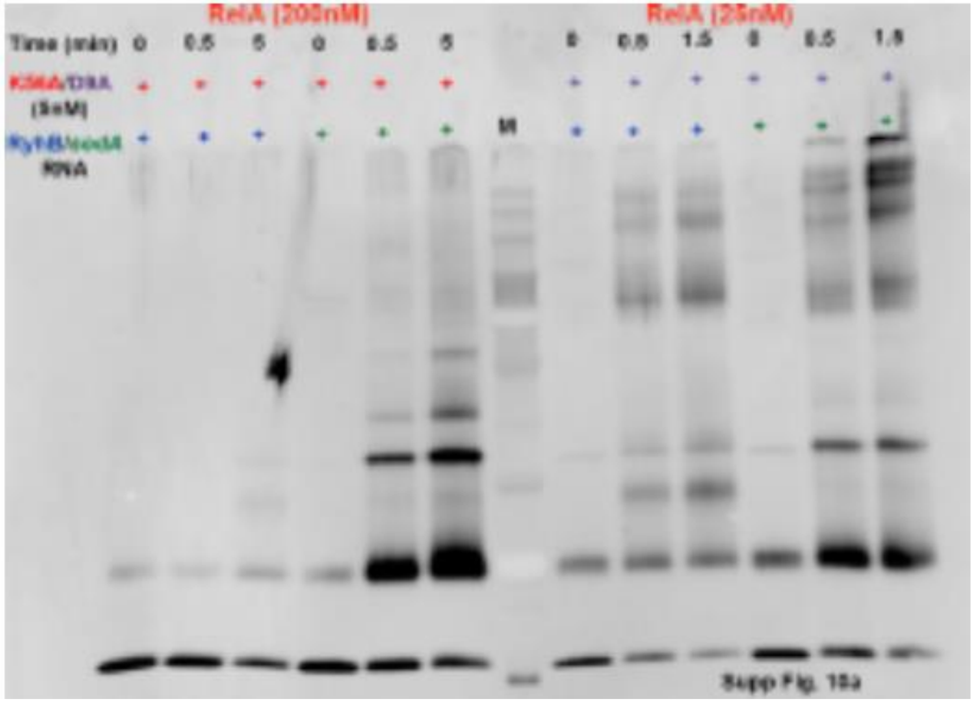

10a
Anti-Hfq antibody

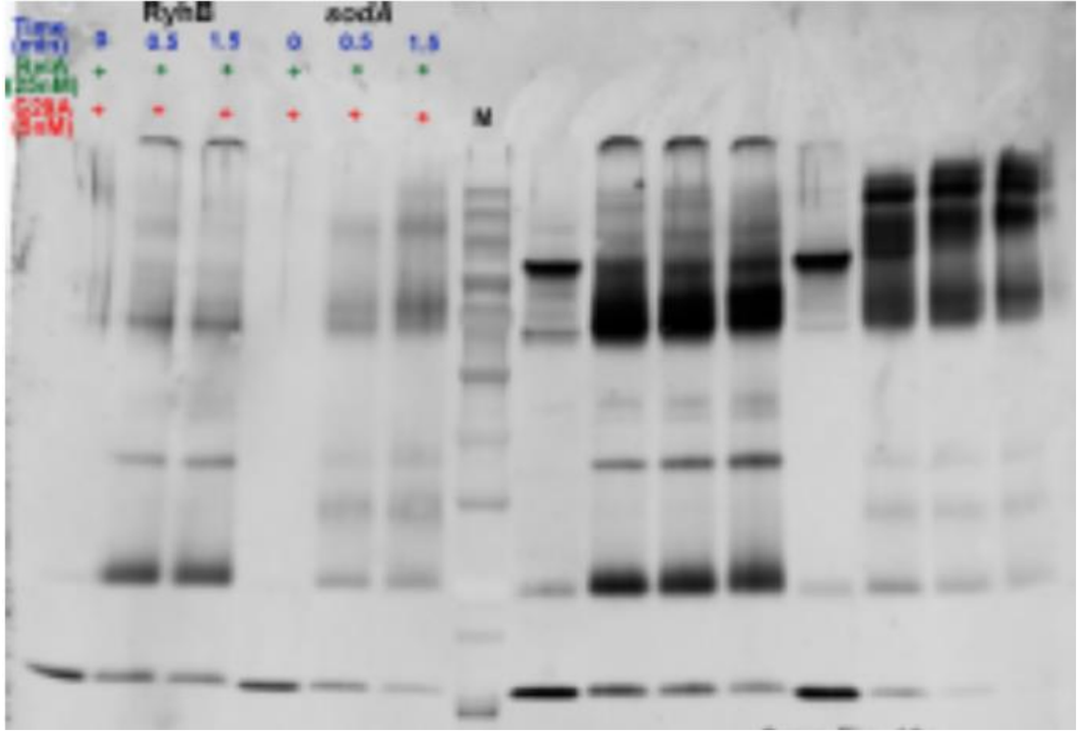

10c
Anti-Hfq antibody

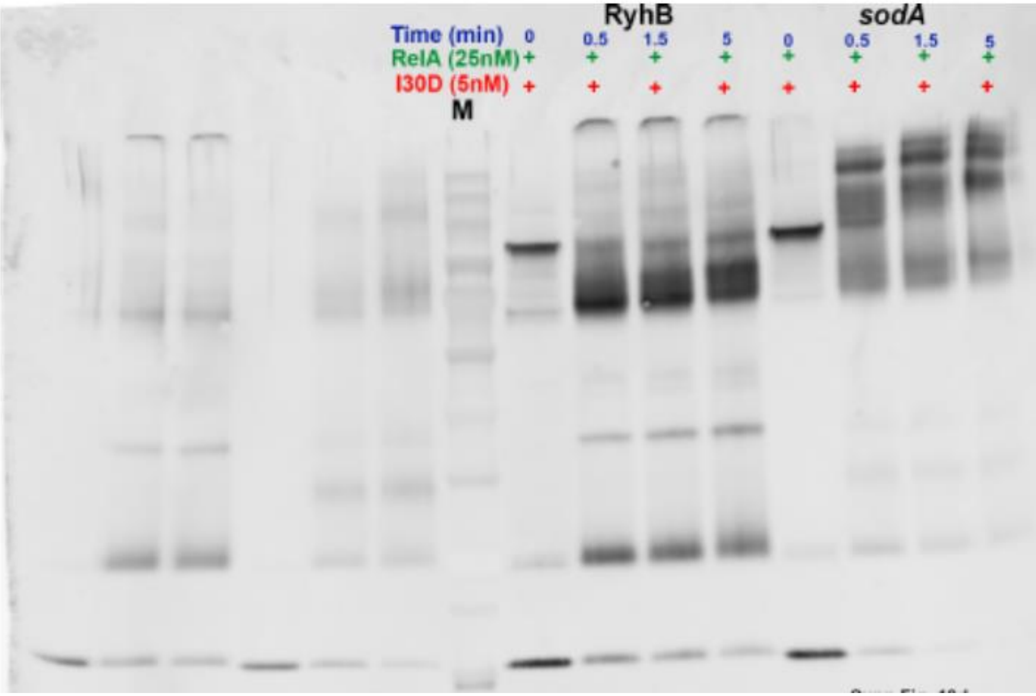

10e
Anti-Hfq antibody

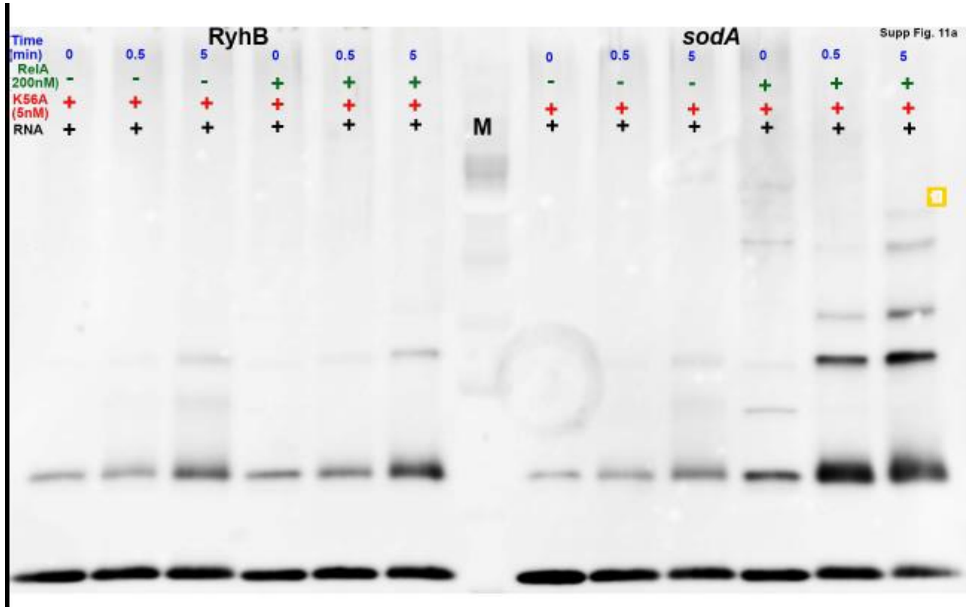

11a
Anti-Hfq antibody

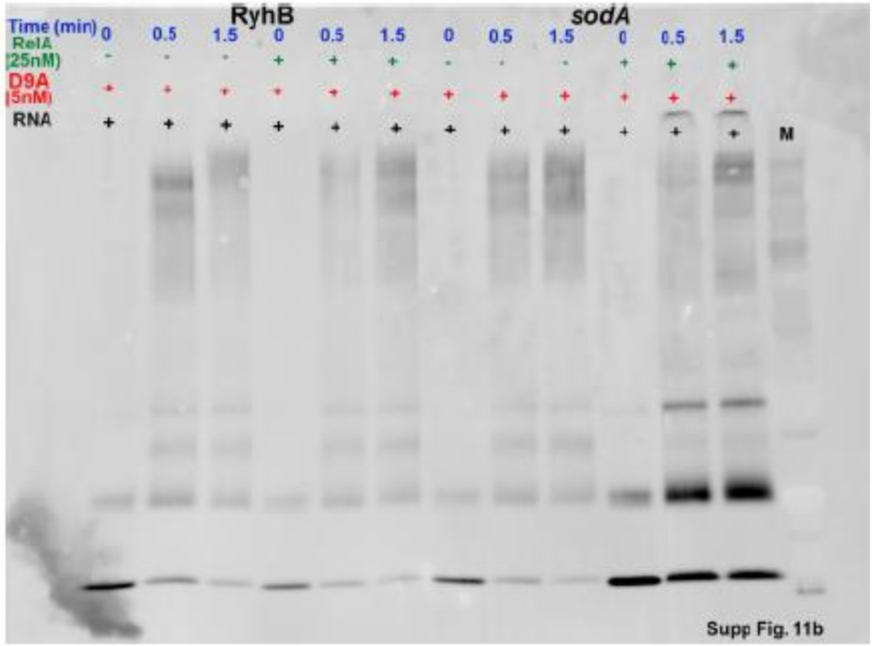

11b
Anti-Hfq antibody

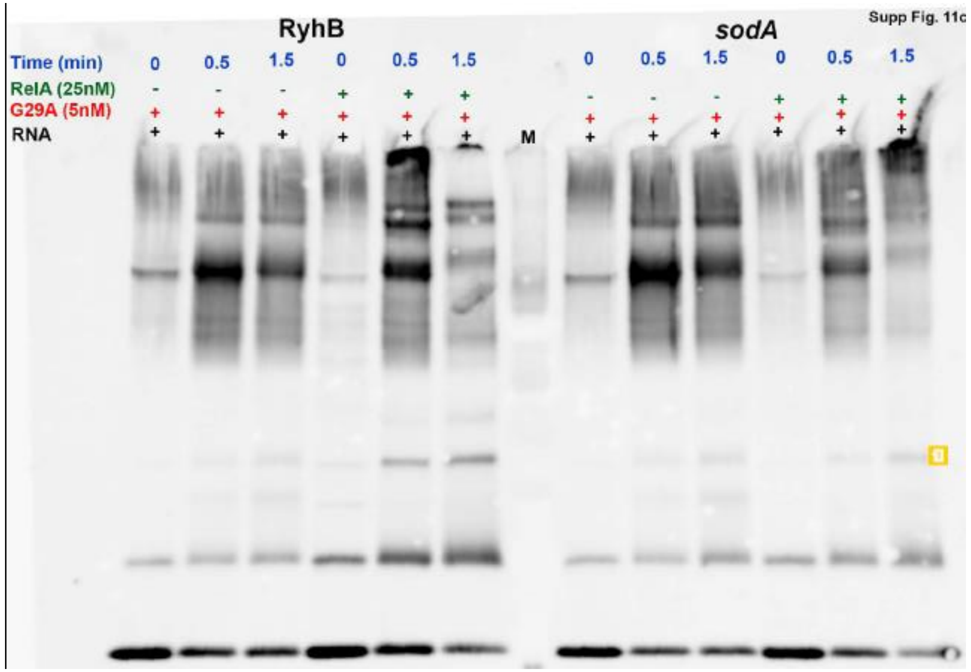

11c
Anti-Hfq antibody

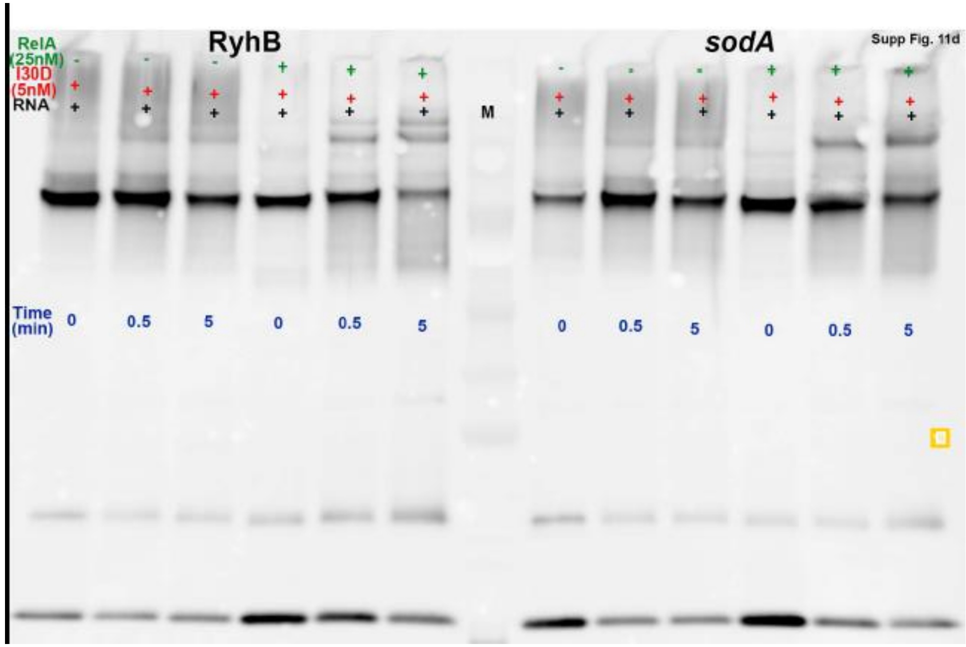

11d
Anti-Hfq antibody

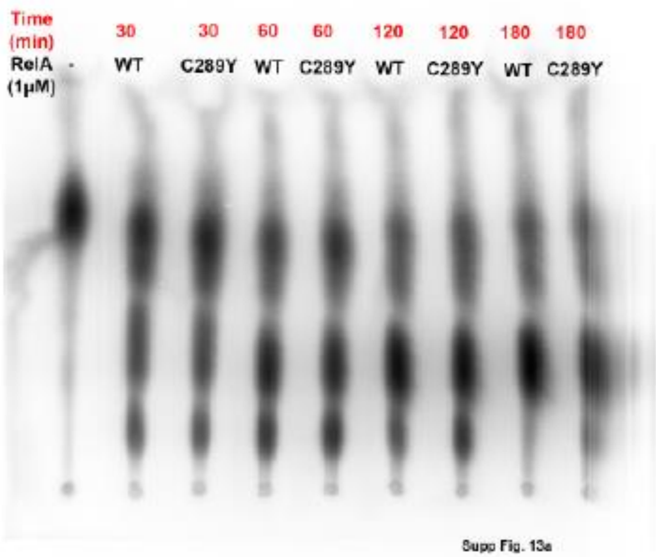
Time (min)
RelA (1µM)
30 30 60 60 120 120 180 180
- WT C289Y WT C289Y WT C289Y WT C289Y
Supp Fig. 13a

13a

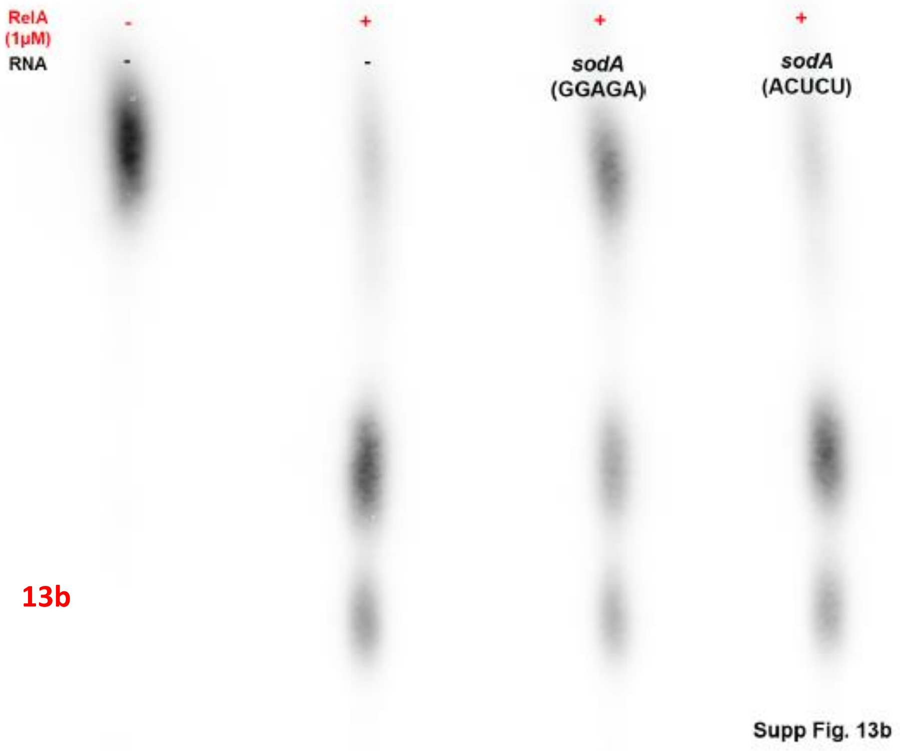
RelA (1µM)
RNA
- + + +
- - *sodA* (GGAGA) *sodA* (ACUCU)
Supp Fig. 13b

13b

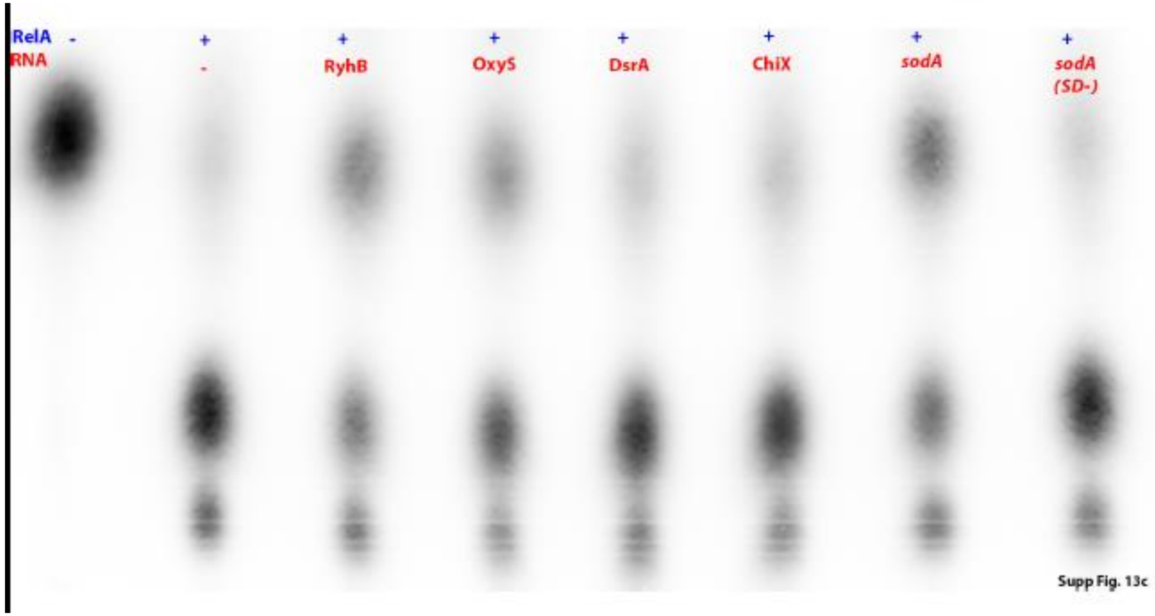
RelA
RNA
- + + + + + + +
- RyhB OxyS DsrA ChiX *sodA* *sodA (SD-)*
Supp Fig. 13c

13c

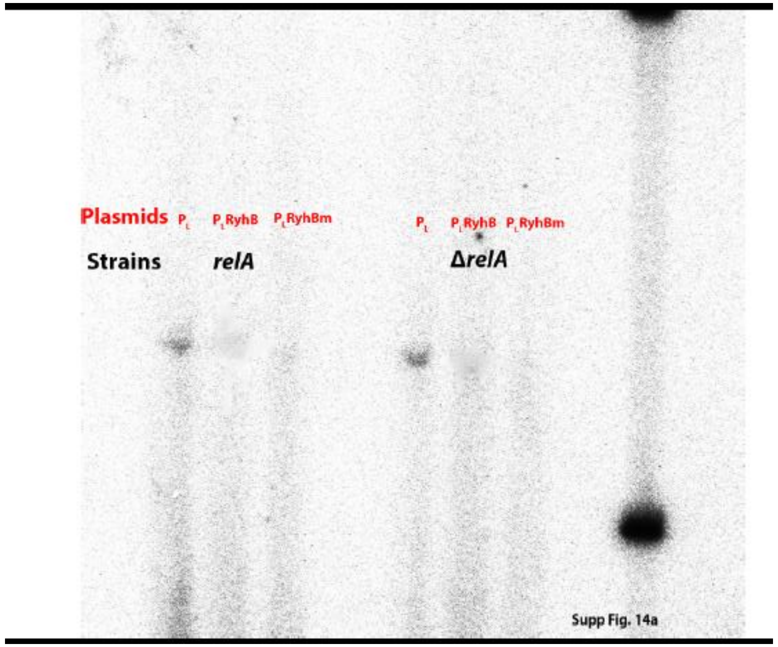

Supp Fig. 14a

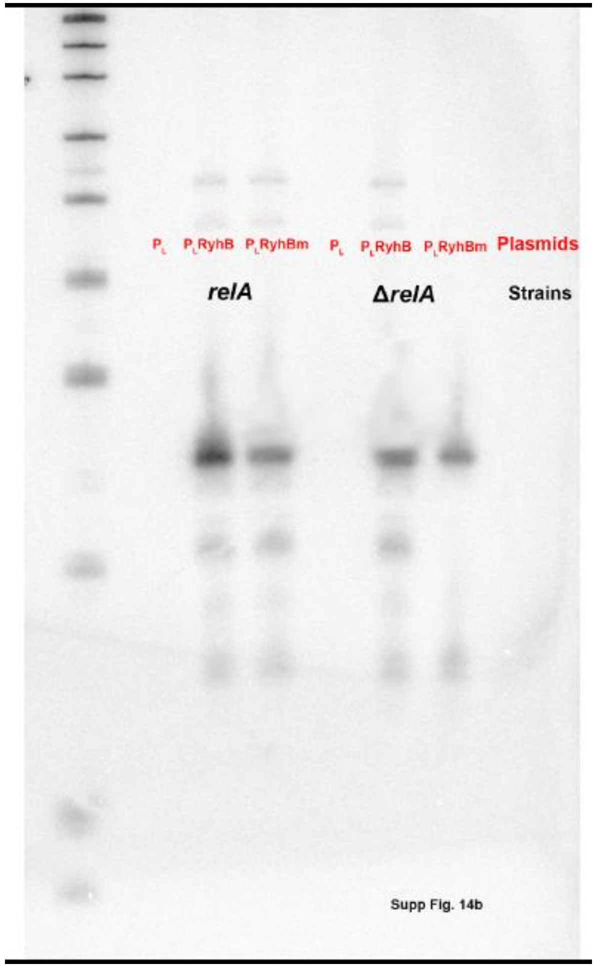

Supp Fig. 14b

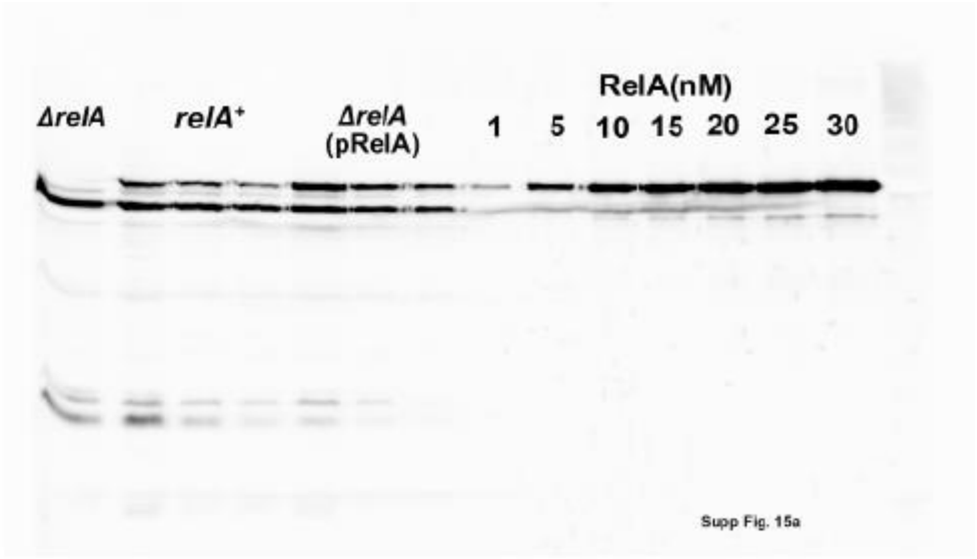

15a
Anti-RelA antibody

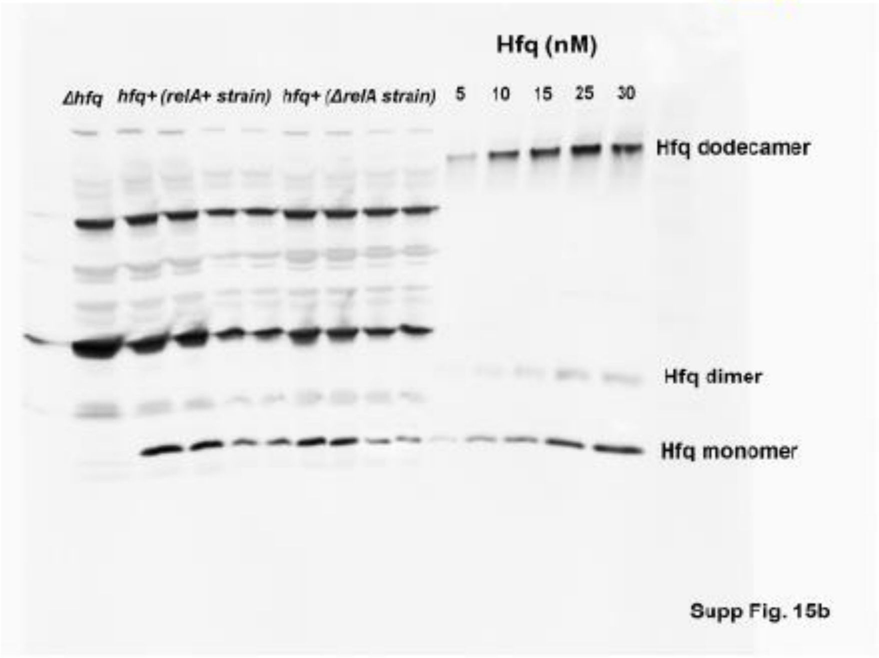

15b
Anti-Hfq antibody

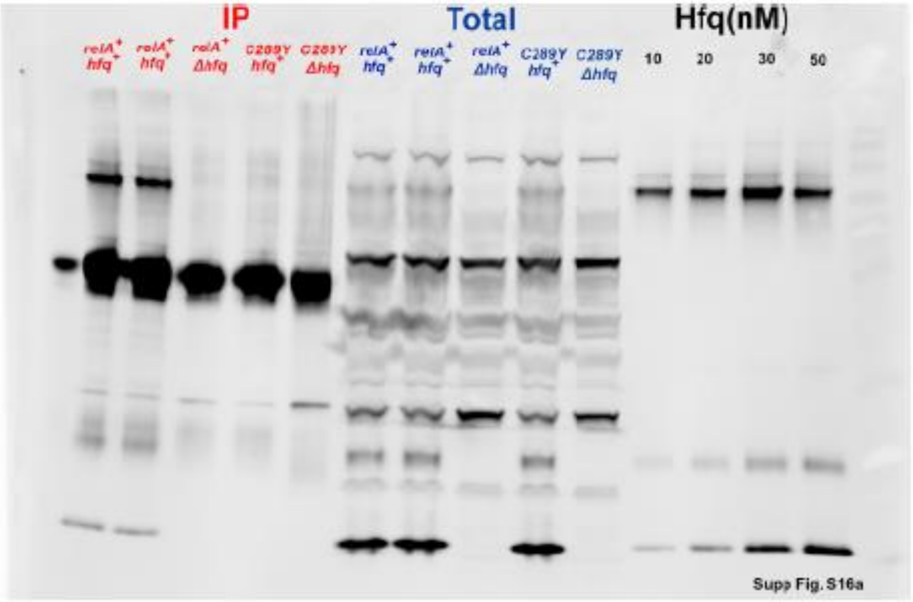

16a
Anti-Hfq antibody

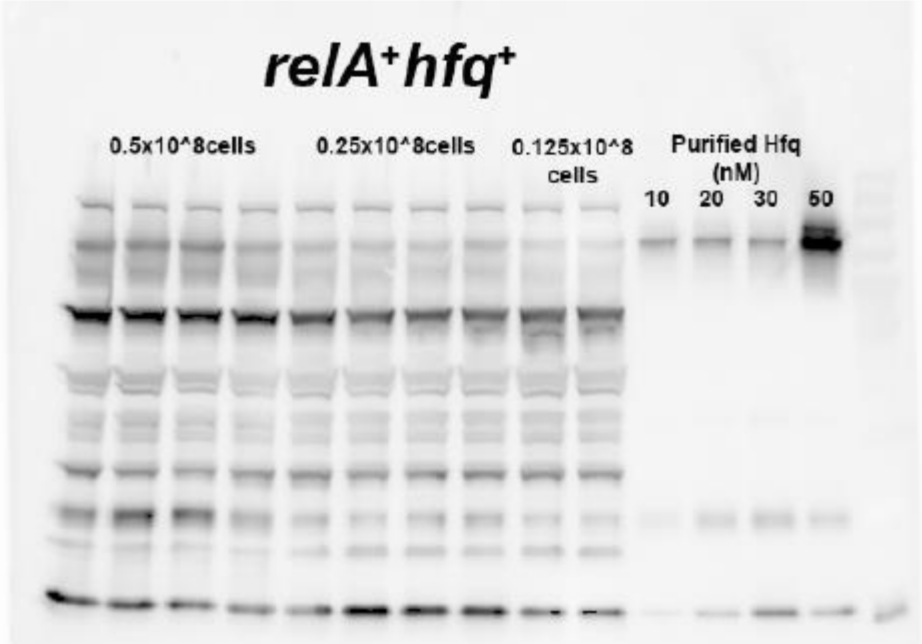

16b
Anti-Hfq antibody

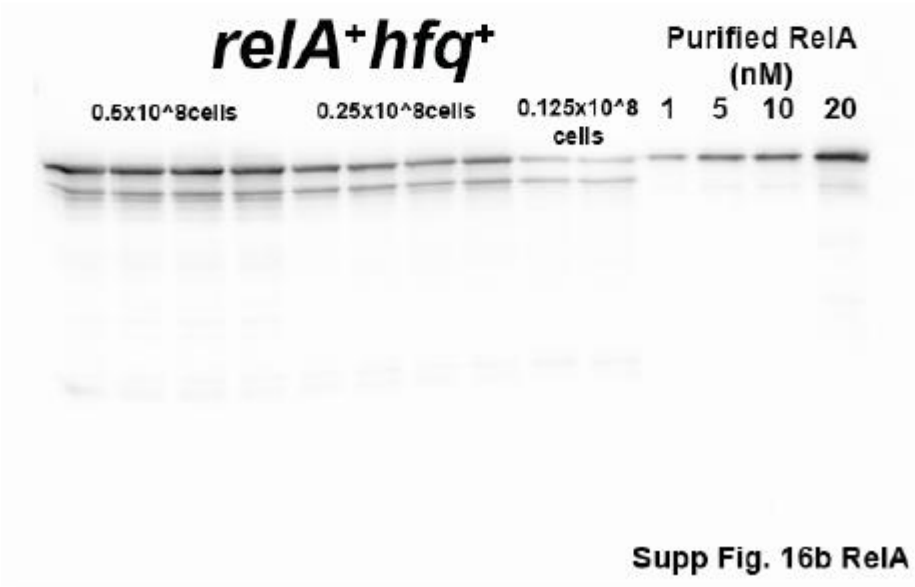

16b
Anti-RelA antibody
